# Physical activity as a protective factor for dementia and Alzheimer’s disease: systematic review, meta-analysis and quality assessment of cohort and case–control studies

**DOI:** 10.1136/bjsports-2021-104981

**Published:** 2022-03-17

**Authors:** Paula Iso-Markku, Urho M Kujala, Keegan Knittle, Juho Polet, Eero Vuoksimaa, Katja Waller

**Affiliations:** 1 Institute for Molecular Medicine Finland (FIMM), HiLIFE, University of Helsinki, Helsinki, Finland; 2 HUS Diagnostic Center, Clinical Physiology and Nuclear Medicine, University of Helsinki and Helsinki University Hospital, Helsinki, Finland; 3 Faculty of Sport and Health Sciences, University of Jyväskylä, Jyväskylä, Finland

**Keywords:** physical activity, public health, cohort studies, neurology

## Abstract

**Objective:**

Physical activity (PA) is associated with a decreased incidence of dementia, but much of the evidence comes from short follow-ups prone to reverse causation. This meta-analysis investigates the effect of study length on the association.

**Design:**

A systematic review and meta-analysis. Pooled effect sizes, dose–response analysis and funnel plots were used to synthesise the results.

**Data sources:**

CINAHL (last search 19 October 2021), PsycInfo, Scopus, PubMed, Web of Science (21 October 2021) and SPORTDiscus (26 October 2021).

**Eligibility criteria:**

Studies of adults with a prospective follow-up of at least 1 year, a valid cognitive measure or cohort in mid-life at baseline and an estimate of the association between baseline PA and follow-up all-cause dementia, Alzheimer’s disease or vascular dementia were included (n=58).

**Results:**

PA was associated with a decreased risk of all-cause dementia (pooled relative risk 0.80, 95% CI 0.77 to 0.84, n=257 983), Alzheimer’s disease (0.86, 95% CI 0.80 to 0.93, n=128 261) and vascular dementia (0.79, 95% CI 0.66 to 0.95, n=33 870), even in longer follow-ups (≥20 years) for all-cause dementia and Alzheimer’s disease. Neither baseline age, follow-up length nor study quality significantly moderated the associations. Dose–response meta-analyses revealed significant linear, spline and quadratic trends within estimates for all-cause dementia incidence, but only a significant spline trend for Alzheimer’s disease. Funnel plots showed possible publication bias for all-cause dementia and Alzheimer’s disease.

**Conclusion:**

PA was associated with lower incidence of all-cause dementia and Alzheimer’s disease, even in longer follow-ups, supporting PA as a modifiable protective lifestyle factor, even after reducing the effects of reverse causation.

## Introduction

Worldwide, around 50 million people suffer from dementia. This number is projected to triple by 2050, with two-thirds of these people living in low-income and middle-income countries.^1^ The economic burden of dementia is estimated to be as high as US$818 billion annually,^2^ thus making dementia prevention a health priority in ageing societies. Physical inactivity is one of 12 potentially modifiable risk factors suggested to account for about 40% of old-age dementias.^3^ Several pathways through which physical activity (PA) may prevent dementias have been proposed: decreased production of β-amyloid, increased removal of β-amyloid, improved brain vasculature and blood flow, and antioxidative and inflammatory processes in the brain,^4^ as well as indirect pathways through improvements in sleep, mood and other cardiovascular risk factors.

Meta-analyses indicate an association between PA and decreased risk of all-cause dementia,^5–7^ including in a dose–response manner.^8^ However, many previous meta-analyses lack rigorous quality assessments,^7^ and the association between PA and dementia appears absent when PA is measured before the age of 65^7 8^ or in follow-ups longer than 10 years.^6 9^ As the Alzheimer’s disease process starts decades before diagnosis,^10^ even studies with 10-year follow-ups are likely to include participants with preclinical Alzheimer’s disease. Thus, studies assessing mid-life PA and old-age dementia diagnoses with a follow-up of at least 20 years are needed to confirm whether PA is a modifiable protective lifestyle factor of dementia.

This systematic review and meta-analysis examines if mid-life PA is a protective factor of all-cause dementia, Alzheimer’s disease and vascular dementia. We examine separately studies with follow-ups longer than 20 years, high-quality studies and studies with younger cohorts to reduce the effect of reverse causality. Because the association of PA and dementia might potentially be modified by the apolipoprotein E (ApoE) genotype,^11–13^ education,^14^ PA type,^12 15^ sample size^16^ and funding source,^17^ we additionally examine these factors as possible moderators of PA–dementia associations.

## Methods

This systematic review and meta-analysis is reported in line with the Preferred Reporting Items for Systematic Reviews and Meta-Analyses statement^18^ (see online supplemental material part 1) and was registered on PROSPERO (CRD42018083236). However, due to insufficient data, some registered analyses were not conducted and the original registered plan was adapted (online supplemental material part 1).

10.1136/bjsports-2021-104981.supp1 Supplementary data

### Eligibility criteria

#### Types of studies

We included prospective cohort studies and case–control studies with a baseline measure of PA and a follow-up measure of all-cause dementia, Alzheimer’s disease or vascular dementia. Only studies with follow-ups longer than 1 year were included.

#### Types of participants

Participants were adults (≥20 years of age at baseline). We excluded studies where participants had some specific disease at baseline or where the cohort had established dementia or mild cognitive impairment at baseline. For populations that were older than mid-life (defined as mean or median age <55 years and maximum age <65 years or mean age plus 1 SD <60 years), a valid measure of baseline cognition was required to be reported. This was done to minimise the possibility of including cohorts with a prodromal state of dementia and to account for the long preclinical period of Alzheimer’s disease^10^ and the typical age of dementia onset.^19^

#### Types of exposure

We included studies assessing PA with objective measures or questionnaires. We excluded studies examining single bouts of PA, retrospectively reported PA, fitness levels or PA levels measured extending over the follow-up period.

#### Types of outcomes

Studies needed to report the association between PA and all-cause dementia, Alzheimer’s disease or vascular dementia. We included studies that diagnosed dementia based on valid measures of cognition or register data, but excluded studies that based dementia diagnosis on cause of death data in more than 50% of the participants.

#### Types of reports

Full-text reports in English were included.

The decision rules that supplement these inclusion and exclusion criteria are described in online supplemental material part 1.

### Search strategy

We conducted a systematic literature search in six electronic databases (PubMed, CINAHL, Scopus, PsycInfo, SPORTDiscus and Web of Science). Two reviewers conducted searches in all six databases, with the last search undertaken on 26 October 2021. The keywords of the original search included physical activity, physically active, sport, athletics, athlete, running, walking, physical training, dementia, Alzheimer’s disease, Alzheimer’s, cognition, cognitive, executive function, TELE (telephone assessment of dementia), TICS (Telephone Interview of Cognitive Status), MMSE (Mini-Mental State Examination), 3-MS (the Modified Mini-Mental State Examination), memory, processing speed, verbal fluency, semantic fluency, reasoning, delayed recall, prospective, longitudinal, follow-up, follow up, observational and cohort. In addition to the search results, individual studies known to the authors were added to the meta-analysis. Further details and example searches are described in the online supplemental material part 1.

### Study selection

Inclusion was based on the assessments of two independent reviewers (PI-M+KW/JP/KK). Disagreements were discussed, and if consensus was not reached, a third independent researcher made the inclusion decision (UMK). Study screening was done in two phases: clearly irrelevant studies were excluded in the title and abstract phase, and thereafter, full-text manuscripts were reviewed. In cases where multiple studies reported similar outcome data from the same cohort, we only included the study with the best quality score, longest follow-up or largest sample size (in this order). Two studies that we excluded from the main meta-analyses (due to other reports from the same cohort being of a higher quality) were however included in the ApoE ɛ4 interaction analysis, as the studies included in the main meta-analyses from these same cohorts did not present any ApoE ɛ4 interaction analyses.^20 21^

### Quality assessment

We developed a quality assessment tool specifically for this systematic review and meta-analysis to provide high transparency of the assessment and to account for the precise characteristics of the addressed study questions (see online supplemental material part 1). The new quality assessment tool assesses and scores the representativeness of the exposed cohort, PA assessment methods, demonstration that dementia was not present at start of study, methods used to control for confounders, outcome assessment methods, length of follow-up and loss to follow-up. We used three existing quality assessment tools to inform the development of our quality assessment tool: the Newcastle-Ottawa Quality Assessment Form for Cohort Studies,^22^ the performance bias estimator by Shiri and Falah-Hassani^23^ and the quality assessment tool for quantitative studies from the Effective Public Health Practice Project Quality Assessment.^24^

Two researchers reviewed the studies with the quality assessment tool independently (PI-M+KW/JP). Disagreements were resolved with discussion. If the study cited other papers, at maximum three papers were sought for the required information. We used a quality scoring system with three categories based on the assumption that studies of high quality have less possibility of reverse causation, the study cohort is not selected and the measurement of both dementia and PA is valid (good quality: ≥2.5+1+≥2.5 stars, moderate quality: ≥2+≥0.5+≥2 stars, poor quality: not reaching good or moderate quality).

### Data extraction

The following outcomes and moderator data were extracted from the included studies: rates of all-cause dementia, Alzheimer’s disease and vascular dementia incidence; PA levels; estimates of the associations between PA levels and all-cause dementia, Alzheimer’s disease or vascular dementia; length of follow-up; sample age and gender make-up; sample size; country of origin; publication year; study design (including a twin study or not); work-related or leisure-time PA; confounders (age, cognition at baseline, chronic diseases, education, gender, vascular risk factors, ApoE ɛ4); follow-up and participation rate; gender interaction; stratification of results according to gender; ApoE ɛ4 interaction results; results stratified according to ApoE ɛ4 allele; number of adjusted confounders; study quality and funding (online supplemental material part 2). Two reviewers extracted the estimates of association (PI-M+KW/JP) and follow-up length (PI-M+KW). The likeness of the extractions was compared and disagreements were resolved by discussion. The estimates with the best quality assessment scores and the most extensive adjustments were included. For example, if baseline cognition was only measured and controlled for in one subgroup of the study sample, then data for that subgroup were extracted instead of the uncontrolled data from the full sample. Studies using the WHO PA recommendation^25^ as the category cut-off were also preferred if many estimates were presented. The data extraction of moderators other than follow-up length was done by one reviewer (PI-M).

10.1136/bjsports-2021-104981.supp2 Supplementary data

Two researchers (PI-M and KW) independently assessed whether the PA categories and reference categories in each study met the WHO PA recommendation.^25^ Disagreements were discussed until consensus was reached.

### Patient involvement

This meta-analysis combines data from pre-existing data sets. No patients were involved in study design, planning the search strategies, planning the quality assessment or sensitivity analyses, implementation of the study, interpretation of the results or writing up the results.

### Statistical analyses

Summary statistics were relative risks (RRs) with 95% CIs. For studies that did not report RR data, ORs or HRs were converted into RRs. OR data were converted to RRs using the formula RR=OR/(1−p0+p0*OR), when the outcome occurred in less than 10% of the sample, with p0=outcome incidence in the whole study population.^26^ When the outcome was common (>10%), we used the square root transformation of OR as recommended by VanderWeele.^27^ We transformed HRs into RRs using the following formula: $RR=(1-e^{\wedge }(HRxln(1-r)))/r$ , where r is the incidence rate of dementia for the reference group.^28^ A separate RR was calculated for each higher PA category reported in the included study by comparing each higher PA category to the lowest PA level in the study (eg, an inactive or reference category).

For the main meta-analyses, we pooled all estimates of the relationship between PA and all-cause dementia, Alzheimer’s disease and vascular dementia, combining categorical and continuous measures of PA. We used a random-effects model with inverse-variance as the weighting method and estimated the statistical heterogeneity with DerSimonian-Laird method (indexed with the I^2^ value). We conducted sensitivity analyses to examine the impacts of removing the study with largest sample size and highest weight on the overall result. An additional analysis examined this relationship within high-quality studies that had measured PA in mid-life and had a follow-up longer than 20 years.

Meta-regressions and comparative subgroup analyses examined the effects of baseline age, follow-up length and meeting the WHO PA recommendation on the association of PA and all-cause dementia, Alzheimer’s disease and vascular dementia. Studies for which the reference category exceeded the WHO guidelines were excluded from the analysis of meeting the PA recommendations.

Next, we performed planned sensitivity analyses to examine the effects of sample size, PA type (leisure time or both leisure time and work related) and other covariates on the relationships of PA and all-cause dementia, Alzheimer’s disease and vascular dementia. Additionally, we examined the effect of funding source (no commercial funding source vs at least one commercial funding source) on the associations. There were too few twin studies and studies addressing gender effects to conduct the prespecified sensitivity analyses of these issues. Finally, we examined only the highest PA level compared with the lowest PA level as has been done in earlier meta-analyses.^5 6 29^

Dose–response meta-analyses were performed to explore linear, quadratic and restricted cubic spline trends between PA levels and RRs of dementia onset. These were conducted in R with the ‘dosresmeta’ package.^30^ A full description of the dose–response methods is available in online supplemental material part 3.

10.1136/bjsports-2021-104981.supp3 Supplementary data

An additional preplanned sensitivity analysis was performed to examine if the presence of ApoE ε4 allele moderates the associations between PA and all-cause dementia, Alzheimer’s disease and vascular dementia. Pooled estimates for the association of PA and all-cause dementia, Alzheimer’s disease or vascular dementia were calculated separately for ApoE ε4 carriers and non-carriers, and a significance test compared the results across the two subgroups.^31^

Funnel plots were used to examine the potential publication bias. Primary analyses were conducted in Stata V.16.0 (StataCorp).

## Results

**Figure 1:**
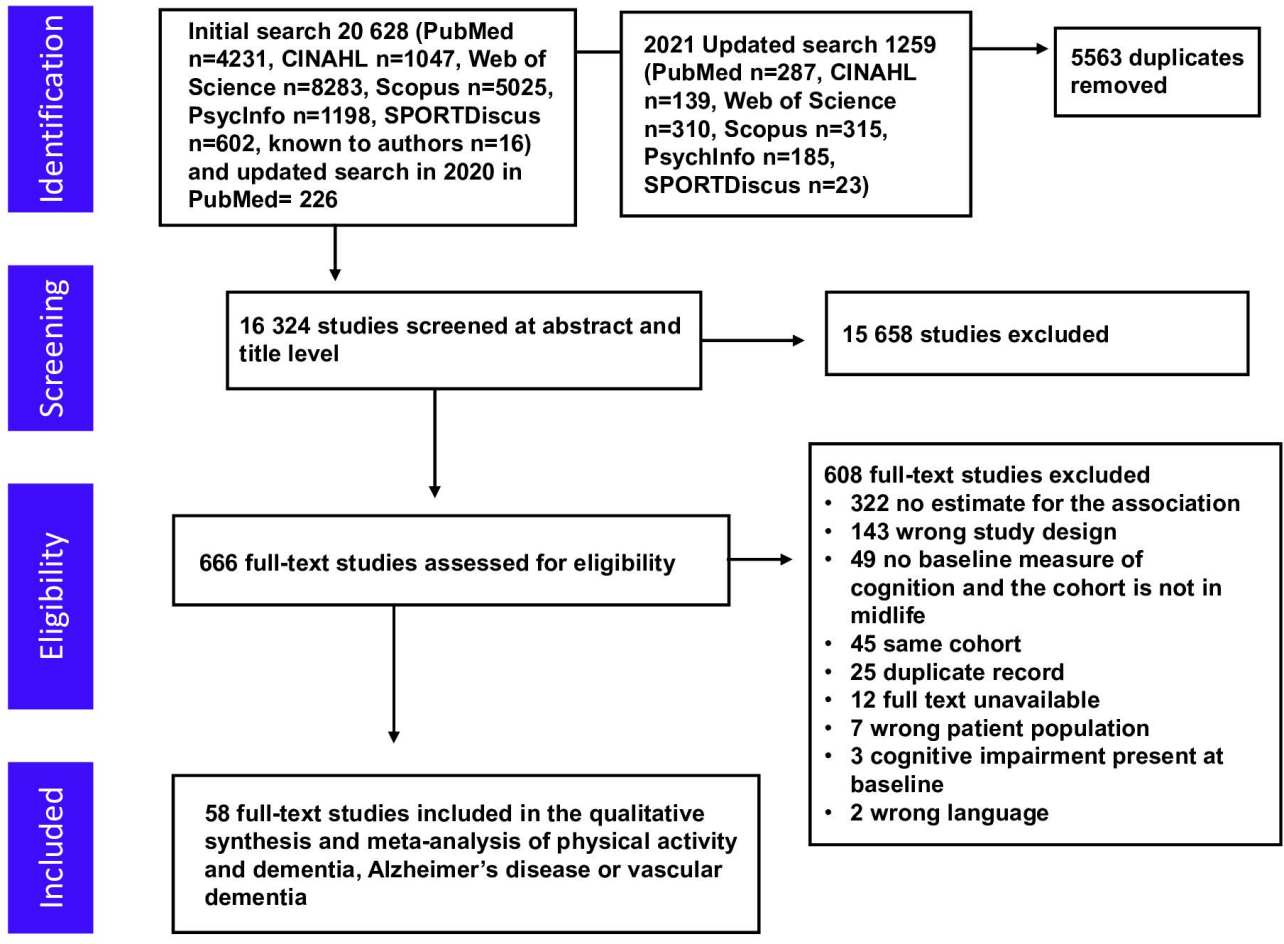
Flow diagram showing the screening process and the search results.

Database searches identified 16 324 articles, of which 15 658 were excluded based on title and abstract screening (figure 1). We assessed 666 full-text articles, 58 of which reported a study that fulfilled the inclusion criteria.^9 11 12 15 32–85^ Overall, studies included 257 983 (range: 67–81 087), 128 261 (range: 300–71 157) and 33 870 (range: 638–20 639) participants for all-cause dementia, Alzheimer’s disease and vascular dementia outcomes, respectively.

### Methodological quality

Methodological quality is reported in online supplemental material part 2. The number of studies of high quality was very low (four all-cause dementia studies,^9 40 55 61^ three Alzheimer’s disease studies^9 12 40^ and one vascular dementia study).^40^ Selection, study length and follow-up rate were the most problematic domains of study quality, with 62%, 65% and 41% of studies receiving the lowest rating on these three quality domains, respectively.

#### PA and all-cause dementia

**Figure 2:**
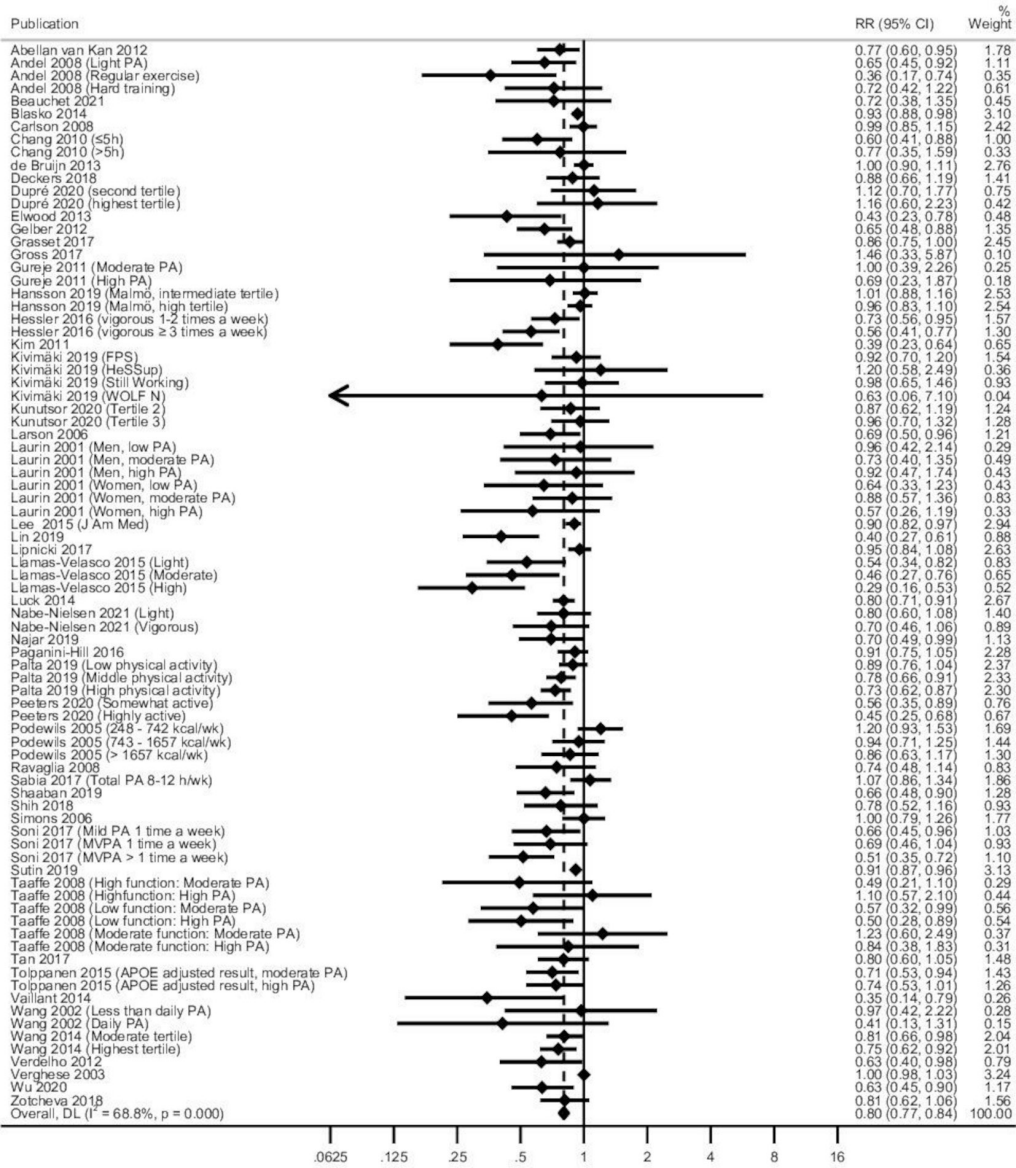
Longitudinal observational studies of physical activity (PA) and all-cause dementia: forest plot. APOE, apolipoprotein E; MVPA, moderate to vigorous physical activity; RR, relative risk.

The mean incidence of all-cause dementia was 10.9% (total n in the analyses=257 983). When compared with the lowest PA category, the pooled RR in higher PA categories showed an association with a reduced risk of all-cause dementia (RR 0.80, 95% CI 0.77 to 0.84) (figure 2, table 1). Mean follow-up length was 12.9 years (SD 9.5), and mean baseline age was 67.0 (SD 12.9) years. There was substantial heterogeneity between the studies (I^2^=68.7%), but neither baseline age, the length of follow-up nor study quality modified the association significantly (table 1). The result was similar within the 16 studies with at least 20 years of follow-up (RR 0.79, 95% CI 0.71 to 0.87, mean baseline age 50.5 (SD 7.8) years, mean follow-up 27.6 (SD 5.1) years and percentage of participants with dementia at follow-up 14.6%). In four high-quality studies, the pooled RR was 0.82 (95% CI 0.67 to 0.99), with a mean baseline age of 48.2 (SD 3.5) years and mean follow-up of 23.2 (SD 4.5) years and 7.6% of participants with dementia at follow-up. This was very similar to the pooled RR of 0.80 in all studies (table 1).

**Table 1:** PA and all-cause dementia: main results, main sensitivity analyses with meta-regressions and subgroup analyses, and dose–response analysis

	Pooled RR	95% CI	I^2^ (%)	Studies combined (n)	Beta estimate*	95% CI
**All physical activity**	0.80	0.77 to 0.84	68.7	49		
Baseline age (continuous)					1.00	0.98 to 1.02
Baseline age (categorical)						
Age group 30–55 years^9 33 35 36 39 40 42 55 61 62 66 73 77 80^	0.79	0.71 to 0.87	42.9	14		
Age group 55–69 years^44 45 57 58 81 83^	0.82	0.74 to 0.90	70.8	6		
Age group ≥70 years^11 32 34 37 38 41 43 46–54 56 59 60 63–65 72 74–76 79 82^	0.80	0.75 to 0.85	70.4	29		
Follow-up length (continuous)					1.00	0.97 to 1.03
Follow-up length (categorical)						
Follow-up length <5 years^38 43 46 51 52 54 63 75 78 79^	0.61	0.50 to 0.74	64.8	10		
Follow-up length 5–20 years^9 11 32 34 37 41 44 45 47–50 56–59 64 65 72 74 76 81–83^	0.86	0.82 to 0.90	64.2	24		
Follow-up length ≥20 years^9 33 35 36 39 40 42 53 55 60–62 66 73 77 80^	0.79	0.71 to 0.87	44.8	16		
Study quality (high vs moderate vs low)†					0.99	0.64 to 1.53
Low quality^9 11 32 34 35 38 41 43–47 49–51 56–58 60 62–66 72 74–76 78 79 81 83^	0.81	0.77 to 0.85	75.5	32		
Moderate quality^9 33 36 37 39 42 48 52–54 59 73 77 80 82^	0.79	0.72 to 0.86	28.4	15		
High quality^9 40 55 61^	0.82	0.67 to 0.99	58.9	4		
Meeting PA guidelines‡ 11 33 36 38 43 44 48 53–55 59–61 63 65 72 73 76 80 83	0.82	0.76 to 0.87	22.0	20		
Not meeting PA guidelines‡ 9 11 32 33 36 38 44 45 47–49 51 57 58 61 62 65 66 72–75 77–79 83	0.76	0.69 to 0.83	60.8	25		
Highest quality studies only: age group 30–55 years, follow-up length >20 years and high quality^40 55 61^	0.79	0.62 to 1.01	67.4	3		

*Beta estimate is the regression coefficient from the meta-regression examining the relationship of modifier or continuous PA on the log risk ratio of dementia.

†Study quality was assessed with a quality assessment tool we developed (see online supplemental material part 1 for details).

‡The test for heterogeneity between groups was non-significant (p=0.202).

I^2^, heterogeneity; PA, physical activity; RR, relative risk.

Only three studies were of high quality, had a young baseline age of 30–55 years and had a follow-up longer than 20 years. The pooled RR in these studies was also similar to the pooled RR in all studies, but not significant (pooled RR 0.79, 95% CI 0.62 to 1.01). Omitting the study with the largest sample size or the study with the largest weight did not significantly change the result (online supplemental table S1). Sample size, funding source, adjusting for ApoE ε4 status, baseline cognition or education did not significantly modify the association of PA and all-cause dementia (online supplemental table S1). The risk of all-cause dementia did not significantly differ between PA levels meeting or not meeting the WHO recommendations of PA (table 1, test for heterogeneity between groups: p=0.202). The two studies examining the association of work-related PA and all-cause dementia showed an opposite trend than other PA (RR 1.25, 95% CI 0.98 to 1.59) (online supplemental table S1).

**Figure 3:**
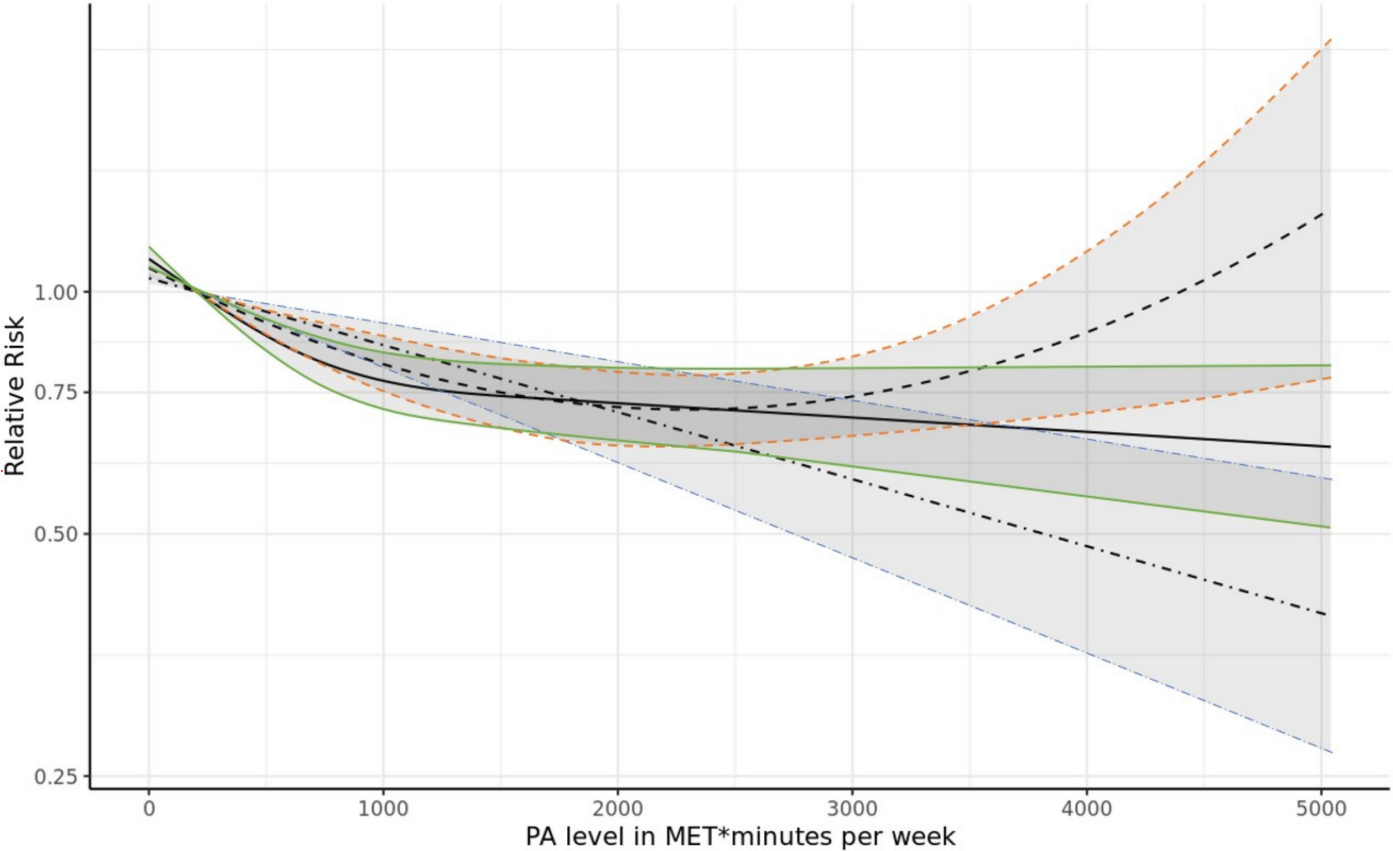
Dose–response analysis of physical activity (PA) levels and all-cause dementia incidence. Linear trend shown with dashed-dotted line and 95% CI in blue; quadratic trend shown with dashed line and 95% CI in orange; and cubic spline trend shown with solid line and 95% CI in green. MET, metabolic equivalent of energy expenditure.

**Figure 4:**
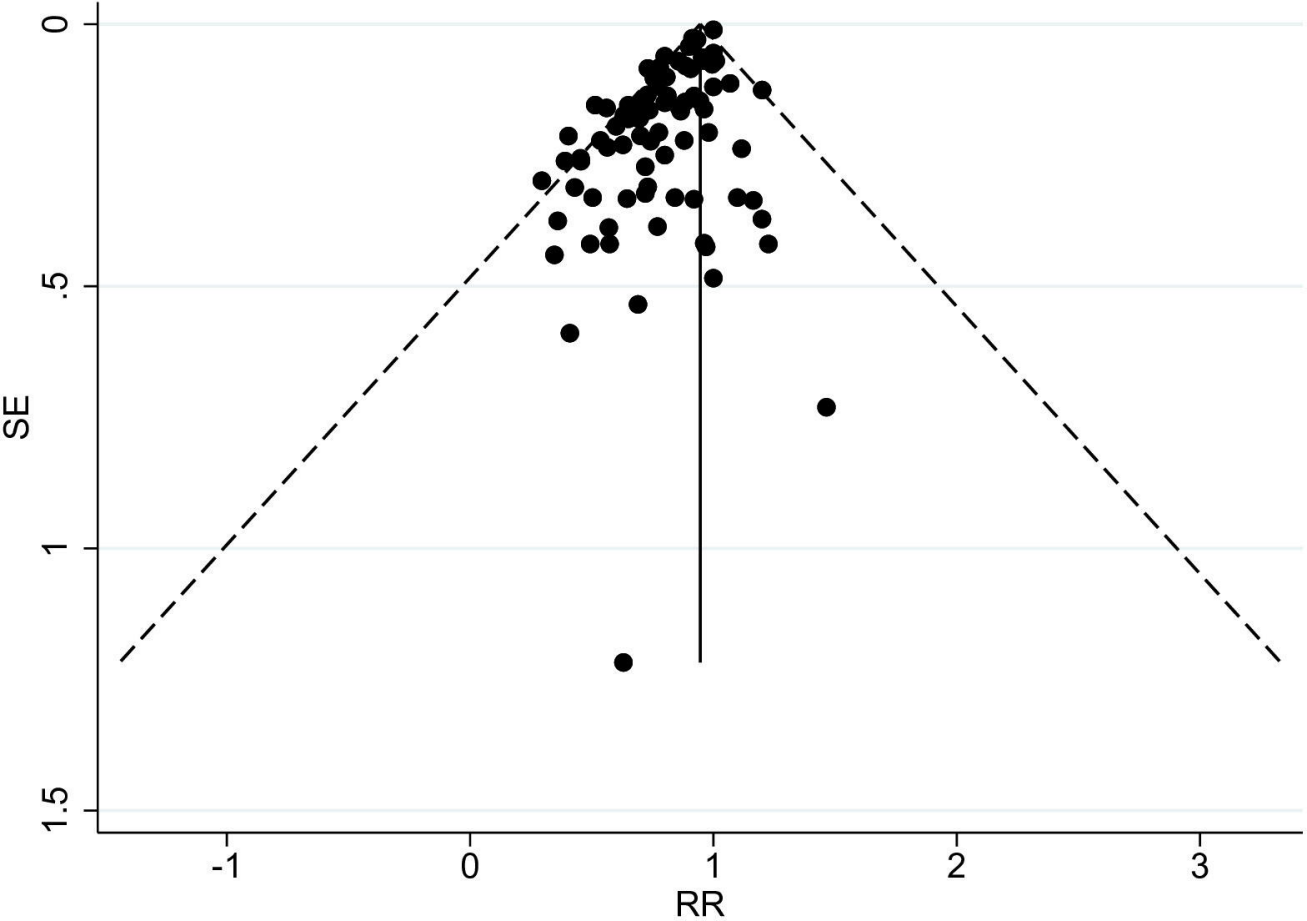
Funnel plot for the longitudinal observational studies on physical activity and all-cause dementia with pseudo-95% CIs. RR, relative risk.

Significant linear, quadratic and cubic spline dose–response relationships were observed between increasing PA levels and lower all-cause dementia incidence (figure 3 and online supplemental material part 3). The funnel plot for studies of PA and all-cause dementia showed some asymmetry suggesting some publication bias (under-reporting of studies with no effect, figure 4).

#### PA and Alzheimer’s disease

The mean incidence of Alzheimer’s disease was 8.3% among 128 261 participants. Compared with the lowest PA category, the pooled RR in higher PA categories showed an association with lower incidence of Alzheimer’s disease (RR 0.86, 95% CI 0.80 to 0.93; online supplemental figure S1 and table 2). The mean follow-up length was 11.5 (SD 8.8) years, and mean baseline age was 68.7 (SD 12.4) years. There was moderate heterogeneity between the studies (I^2^=47.6%), and neither baseline age, the length of follow-up nor study quality modified the association significantly (table 2). This result was similar among the seven studies with at least 20 years of follow-up (RR 0.76, 95% CI 0.64 to 0.90, mean baseline age 52.8 (SD 8.9) years, mean follow-up 26.8 (SD 6.4) years and percentage of participants with Alzheimer’s disease at follow-up 5.2%). Among the three high-quality studies, the pooled RR of 0.71 (95% CI 0.42 to 1.22) was non-significant (table 2). Neither sample size, adjustment for ApoE ε4, baseline cognition nor education significantly moderated the association between PA and Alzheimer’s disease incidence (online supplemental table S2). The risk of all-cause dementia did not significantly differ between PA levels meeting or not meeting the WHO recommendations of PA (table 1, test for heterogeneity between groups: p=0.202).

**Table 2:** PA and Alzheimer’s disease: main results, main sensitivity analyses with meta-regressions and subgroup analyses, and dose–response analysis

	Pooled RR	95% CI	I^2^ (%)	Cohorts combined (n)	Beta estimate*	95% CI
**All PA**	0.86	0.80 to 0.93	47.6	24		
Baseline age (continuous)					1.00	0.97 to 1.03
Baseline age (categorical)						
Age group 30–55 years^9 12 33 40 77 80^	0.81	0.66 to 0.99	37.3	6		
Age group 55–69 years† 44	1.09	0.96 to 1.24	0.0	1		
Age group ≥70 years^11 37 38 47 48 52 54 59 60 63 67–71 84 85^	0.84	0.77 to 0.93	48.5	17		
Follow-up length					1.00	0.96 to 1.04
Follow-up length <5 years^38 52 54 63 70 84^	0.93	0.79 to 1.08	48.4	6		
Follow-up length 5–20 years^9 11 37 44 47 48 59 67–69 71 85^	0.87	0.78 to 0.97	41.3	12		
Follow-up length ≥20 years^9 12 33 40 60 77 80^	0.76	0.64 to 0.90	16.9	7		
Study quality (low vs moderate vs high)‡					1.14	0.59 to 2.22
Low quality^9 11 44 47 60 63 67 68 70 71^	0.97	0.88 to 1.07	34.1	10		
Moderate quality^9 33 37 38 48 52 54 59 69 77 80 84 85^	0.81	0.74 to 0.90	24.0	13		
High quality 9 12 40	0.71	0.42 to 1.22	71.8	3		
Meeting PA guidelines§ 11 12 33 38 44 47 48 54 59 60 63 68 69 71 80 84	0.75	0.64 to 0.88	43.4	16		
Not meeting PA guidelines§ 9 11 33 38 44 48 67 69 77 80	0.94	0.85 to 1.04	0.0	10		
Age group 30–55 years, high quality and follow-up length >20 years^12 40^	0.55	0.29 to 1.03	53.9	2		

*Beta estimate is the regression coefficient from the meta-regression examining the relationship of modifier or continuous PA on the log risk ratio of dementia.

†Only one study, not meta-analytical analysis.

‡Study quality was assessed with a quality assessment tool we developed (see online supplemental material part 1 for details).

§The test for heterogeneity between groups was non-significant (p=0.126).

I^2^, heterogeneity; PA, physical activity; RR, relative risk.

Dose–response meta-analyses revealed a significant cubic spline trend between PA levels and Alzheimer’s disease incidence, but linear and quadratic trends were non-significant (online supplemental material part 3). The funnel plot for studies of PA and Alzheimer’s disease showed some asymmetry suggesting possibly small publication bias (under-reporting of results with no effect, online supplemental figure S2).

#### PA and vascular dementia

The mean incidence of vascular dementia was 3.8% among 33 870 participants. When compared with the lowest PA category, the pooled RR in higher PA categories showed an association with reduced incidence of vascular dementia (pooled RR 0.79, 95% CI 0.66 to 0.95) (online supplemental figure S3 and table S3). Mean follow-up length was 10.9 (SD 8.6) years, and mean baseline age was 67.0 (SD 8.5) years. Statistical heterogeneity between the studies was moderate (I^2^=36.0%).

Neither baseline age, length of follow-up, meeting the WHO PA recommendation, adjusting for baseline cognition nor study quality significantly modified the association (online supplemental tables S3 and S4). There was only one high-quality study with follow-up longer than 20 years and baseline age between 30 and 55 years.^40^ The association between PA and decreased incidence of vascular dementia was significant in this study.

Significant linear, quadratic and cubic spline dose–response relationships between PA and vascular dementia incidence were observed (online supplemental material part 3). The funnel plot for studies of PA and vascular dementia did not suggest publication bias (online supplemental figure S4).

#### ApoE ε4 interaction

Most studies that investigated ApoE ε4 interactions found no significant interactions (9 of 11 studies) (supplementary sensitivity analyses for ApoE ε4 allele). In the studies that reported stratified results according to ApoE ε4 carrier status, the pooled RR between PA and all-cause dementia or Alzheimer’s disease was similar for ApoE ε4 carriers (RR 0.81, 95% CI 0.67 to 0.98) and non-carriers (RR 0.72, 95% CI 0.56 to 0.92). Tests for heterogeneity between groups were invalid because of large heterogeneity between studies (online supplemental figures S5–S8).

## Discussion

This meta-analysis showed that higher PA levels were associated with lower incidence of all-cause dementia, Alzheimer’s disease and vascular dementia. These associations were present for all-cause dementia and Alzheimer’s disease in studies with long follow-ups (>20 years) and in cohorts with baseline age between 30 and 55 years. Neither baseline age nor follow-up length moderated the associations of PA with all-cause dementia or Alzheimer’s disease. Data for vascular dementia were scarce, especially for long follow-ups, but the results supported an inverse association between PA and vascular dementia incidence.

Earlier meta-analyses investigating the associations between PA levels and dementia incidence have been based on short follow-ups,^5 29 86^ and the results have only been significant in studies with short (<10 years) follow-ups^6 9^ or elderly populations.^7 8^ These factors can introduce the possibility of reverse causation, whereby PA levels are affected by dementia.^9^ Participants’ levels of PA may also change during long follow-ups.^87^ In this study, we did not find evidence to suggest that reverse causation or regression dilution bias^88^ affected the observed associations between PA and dementias. Our results therefore support the role of PA as a modifiable protective mid-life lifestyle factor of dementia. However, funnel plots for all-cause dementia and Alzheimer’s disease suggested some publication bias.

In high-quality studies, a non-significant negative association was found between PA and Alzheimer’s disease. However, with only three high-quality studies, the statistical power to show a significant association is low to moderate but the pooled RR estimate in high-quality studies (0.71) was similar to that obtained when including all studies (0.86). Additionally, the meta-regression estimate did not show significant moderation by study quality. Our inclusion criteria were also strict, as we excluded studies with baseline in old age and without a validated measure of cognition at baseline. This procedure reduces the risk of bias due to reverse causality, but led to fewer studies being included in the meta-analysis. The strict representativeness criterion may have unnecessarily limited the number of high-quality studies^88^ and thereby increased the CIs of pooled risk estimates from high-quality studies.

Dose–response meta-analyses showed significant linear, quadratic and cubic inverse associations between PA levels and incidence of all-cause dementia and vascular dementia. The finding of a linear dose–response is in line with the results from Xu *et al* 8; however, in our analysis, the effect of PA on all-cause dementia incidence was greatest when moving from extreme sedentariness to some PA. While a significant cubic spline relationship was observed between PA and Alzheimer’s disease, the model only included two studies that examined the effects of PA levels greater than 1750 MET*min/week. More studies among more physically active cohorts are needed to conclusively determine whether more PAs offer greater protection at the higher end of the spectrum, or whether a moderate level of PA offers similar protective effects.

Our results contrast with those from Kivimäki *et al* 9 who examined individual participant data from many study cohorts worldwide (n=404 840). In that study, no associations were found between PA and all-cause dementia or Alzheimer’s disease when follow-ups were longer than 10 years. Notably, the incidence of all-cause dementia in their meta-analysis was 0.5%. This is an exceptionally low all-cause dementia incidence rate, considering the global annual dementia incidence rate of 17.3% in adults over 60 years of age.^89^ Two factors can explain this. First, the mean age at baseline was 45.5 years in Kivimäki *et al*.^9^ As the mean follow-up length was 14.9 years, the mean age at the end of follow-up was approximately 60.4 years, but the mean age of all-cause dementia diagnosis in the study was 80.6 years. The result was similar in a subanalysis of persons aged 60 years or older.^9^ The follow-up length of over 10 years may also contribute to reverse causation, considering the long preclinical period of Alzheimer’s disease.^10^ Therefore, these earlier results may be susceptible to bias from both early-onset dementias and reverse causation.

The very low all-cause dementia incidence rate in the Kivimäki *et al*’s^9^ study may also be explained by the source of incidence rate data: the meta-analysis used register data (hospitalisations, medical reimbursements and death registers) and a few very large cohorts had only death register data. We excluded studies with dementia mortality as the outcome because the relatively low sensitivity of death registers to detect dementia cases may underestimate its association with risk or protective factors.^90^ Further, dementia mortality in younger individuals is likely to reflect early-onset dementias. On the other hand, there may be a survival bias, whereby those with higher levels of PA live longer and are therefore at an increased risk of developing dementia.

The sensitivity analyses for all-cause dementia and Alzheimer’s disease showed similar estimates among studies that controlled for baseline cognition. Studies have primarily assessed baseline cognition using short screening tests, which are known to result in ceiling effects, and most studies of younger cohorts did not assess cognitive ability at baseline. While many studies collected information on baseline cognition, few adjusted for it in their analyses. Because baseline cognitive ability may be the most robust predictor of cognition later in life,^91^ the absence of rigorous assessments of baseline cognition and failure to control for it in analyses should be seen as major limitations of the included studies and this meta-analysis. Physically active individuals may have higher cognitive reserve to start with,^92^ so studies should examine whether early cognitive ability predicts PA later in life. In many studies investigating PA and other health outcomes, work-related PA shows an inverse association with leisure-time PA when adjusted for socioeconomic status or education.^93 94^ This may also indicate that higher cognitive ability or other unmeasured confounding factors, and not leisure-time PA, may drive the association with a decreased incidence of dementia. Almost all studies in this field have adjusted their results for education level, a widely used proxy for higher cognitive reserve. Still, people with the same number of years of formal education may vary greatly in their cognitive abilities.^95^ Many studies have suggested that ApoE ε4 carrier status modifies the relationship between PA and dementia.^11–13^ Our ApoE ε4 interaction analyses suggest no such modification for all-cause dementia, Alzheimer’s disease or vascular dementia.

### Strengths

This meta-analysis includes extensive data and has examined the association of PA and dementia in longer follow-ups than earlier meta-analyses. Our quality assessment was specifically developed to account for the long preclinical period of dementia, and the quality assessment of PA has been developed in cooperation with sports and exercise medicine experts (KW and UMK). We also studied PA levels meeting a fixed threshold (WHO PA recommendation), and we addressed dose–response relationships between PA with all-cause dementia, Alzheimer’s disease and vascular dementia incidence.

### Limitations

Some limitations of this meta-analysis bear mentioning. Many studies used only rough PA measures (eg, a dichotomous yes or no question to describe exercise participation). These rough measures coupled with the midpoint or mean estimation of MET*min/week PA levels imply that the dose–response meta-analyses likely lack precision, especially when the PA levels within a group were wide. Future cohort studies should use objective or finer grain PA assessments and make individual participant data open whenever possible. Of the included studies, few were high quality, few reported on vascular dementia as an outcome and few had any robust measures of cognition at baseline. The stringent criterion of representativeness may have unnecessarily limited the number of high-quality studies. Additionally, some publication bias may have affected the results for all-cause dementia and Alzheimer’s disease. We searched only studies published in English which is also a possible source of bias.^96^ In addition, the impacts of PA modalities which are themselves associated with increased risk of dementia (eg, boxing)^97^ are not accounted for in the results presented here.

## Conclusions and policy implications

This meta-analysis found inverse associations between PA levels and incidence rates of all-cause dementia and Alzheimer’s disease, even in studies with follow-ups longer than 20 years. This finding supports PA as a modifiable protective lifestyle factor of dementia. Policy makers should continue to promote PA in school and work-life reforms, city planning and health initiatives. However, these conclusions should be tempered slightly, as the meta-analysis was based on observational studies with known limitations compared with intervention studies, as high-quality studies were scarce and some publication bias was present. More research with long follow-ups, adjustment for baseline cognitive performance and valid measures of PA and dementia are needed to confirm these findings. Finally, long-term randomised controlled trials of exercise interventions are needed to establish PA as a causative protective factor for dementia.

**What is already known on this topic?**

- Alzheimer’s disease and related dementias are a major public health concern, and their prevalence is projected to multiply in the coming decades.
- There are no drugs to stop or reverse the dementia process, but lifestyle interventions in mid-life may help delay or prevent dementias.
- Physical inactivity is associated with an increased incidence of dementia, but whether this is due to reverse causation whereby lower physical activity results from the dementia process is under debate.

**What this study adds**

- In this meta-analysis of over 250 000 participants, physical activity was significantly associated with a decreased incidence of all-cause dementia and Alzheimer’s disease, irrespective of follow-up length, baseline age and study quality.
- Physical activity was a protective factor for all-cause dementia and Alzheimer’s disease, even in follow-ups longer than 20 years, suggesting that the association is not simply due to reverse causation.
- Policy makers should support intervention strategies targeting societal increases in physical activity in mid-life, as these may reduce dementia incidence.

## Data Availability

The data used in the analyses of this manuscript have been published alongside with the manuscript as 'Supplementary Material Part 2'.
