# Supplementary material for: Physical activity as a protective factor for dementia and Alzheimer’s disease: systematic review, meta-analysis and quality assessment of cohort and case–control studies
Source: Br J Sports Med. 2022 Mar 17;56(12):701–9. doi: 10.1136/bjsports-2021-104981 (PMC9163715; doi:10.1136/bjsports-2021-104981)
Supplement: Supplementary data [file bjsports-2021-104981supp001.pdf]

Supplementary material: Part 1

Methods

This meta-analysis examines the relationships between PA and the incidence of all-cause dementia, Alzheimer's disease, and vascular dementia. A separate meta-analysis will examine the also pre-registered relationship between PA and cognition outcomes. The study plan was submitted to PROSPERO 2.12.2017 (prior to the first search of the review 11.12.2017) but registration was accepted only after the study implementation (registration 8.1.2018).

The plan of the search has been described in PROSPERO (registration number CRD42018083236). The original searches were done in PsycInfo 11.12.2017, in PubMed 14.12.2017, in CINAHL 19.12.2017, in Web of Science 21.12.2017, in Scopus 21.12.2017, and in SPORTDiscus 15.1.2018 (for search terms, see the search strategy below).

The second search targeted only one database (PubMed) and articles published between 12.12.2017 to 10.12.2020 with reduced search terms ("physical activity", "exercise", "Alzheimer's disease", "cognition", "cognitive decline", "cognitive impairment", and "dementia") using the following filters: Humans, English, Adult 19+, Observational study. The second search was screened by one reviewer alone (PIM).

The third search was better targeted than the excessively wide original search and targeted only dementia. It was conducted in six databases with the search terms: physical activity, sport, walking, physical training, aerobic exercise, dementia, Alzheimer's disease, Alzheimer, prospective, longitudinal, follow-up, follow up, observational, cohort. The sensitivity of this search was tested first for two years from the original search (2015 and 2017) and was found to be good. This better targeted search found 3/3 of the physical activity and dementia articles from the original search from 2015 and 6/7 from the original search from year 2017 (the one study that this search did not find did not mention physical activity in the abstract or title). The searches were done to update the original search 19.10.2021 in CINAHL, 21.10.2021 in PubMed, Web of Science, PsycInfo and Scopus and 26.10.2021 in SPORTDiscus. Below are two search examples first from the original search and then two examples from the third search.

Most studies reported mean or median baseline age but for studies that did not, these were extrapolated. A few studies reported only that the association between physical activity and all-cause dementia, Alzheimer's disease, or vascular dementia was not significant. The authors of these studies were contacted via email to obtain effect size data of these associations, but no requests were fulfilled. Some studies reported the results for sensitivity analyses only partially and if the study quality was better in a partially reported sensitivity analysis, the authors were also contacted. Some of these requests were answered.

The search strategy

The search in CINAHL in 2017

1. physical activity
2. physically active
3. exercise*
4. sport*
5. athletics
6. athlete*
7. run*
8. walk*
9. physical training
10. 1 OR 2 OR 3 OR 4 OR 5 OR 6 OR 7 OR 8 OR 9
11. dementia
12. Alzheimer*
13. Alzheimer's disease
14. cognition
15. cognitive decline
16. cognitive impairment
17. cognitive function
18. executive function
19. TELE
20. TICS
21. MMSE
22. 3-MS
23. memory
24. processing speed
25. verbal fluency
26. semantic fluency
27. reasoning
28. delayed recall
29. 11 OR 12 OR 13 OR 14 OR 15 OR 16 OR 17 OR 18 OR 19 OR 20 OR 21 OR 22 OR 23 OR 24 OR 25 OR 26 OR 27 OR 28
30. prospective
31. longitudinal
32. follow up
33. observational
34. cohort
35. 30 OR 31 OR 32 OR 33 OR 34
36. 10 AND 29 AND 35

Filters: English, Human, All adult

The search in Web of Science in 2017

1. physical activity
2. physically active
3. exercise*
4. sport*
5. athlet*
6. running
7. walking
8. physical training
9. 1 OR 2 OR 3 OR 4 OR 5 OR 6 OR 7 OR 8 OR 9
10. dementia
11. Alzheimer*
12. cognition
13. cognitive
14. executive function
15. TELE
16. TICS
17. MMSE
18. 3-MS
19. memory
20. processing speed
21. verbal fluency
22. semantic fluency
23. reasoning
24. delayed recall
25. 11 OR 12 OR 13 OR 14 OR 15 OR 16 OR 17 OR 18 OR 19 OR 20 OR 21 OR 22 OR 23 OR 24 OR 25 OR 26 OR 27 OR 28
26. prospective
27. longitudinal
28. follow up
29. follow-up
30. observational
31. cohort*
32. 30 OR 31 OR 32 OR 33 OR 34
33. 10 AND 29 AND 35

LANGUAGES: (ENGLISH) AND **[excluding]: RESEARCH AREAS:** (GOVERNMENT LAW OR ASTRONOMY ASTROPHYSICS OR AUTOMATION CONTROL SYSTEMS OR TRANSPLANTATION OR ENERGY FUELS OR ORTHOPEDICS OR SUBSTANCE ABUSE OR NUTRITION DIETETICS OR METEOROLOGY ATMOSPHERIC SCIENCES OR ZOOLOGY OR ENGINEERING OR MATERIALS SCIENCE OR OPTICS OR SURGERY OR WATER RESOURCES OR MATHEMATICS OR COMPUTER SCIENCE OR ROBOTICS OR CRIMINOLOGY PENOLOGY OR CHEMISTRY OR AGRICULTURE OR PHARMACOLOGY PHARMACY OR GEOLOGY OR MARINE FRESHWATER BIOLOGY OR DENTISTRY ORAL SURGERY MEDICINE OR METALLURGY METALLURGICAL ENGINEERING OR VETERINARY SCIENCES OR LINGUISTICS OR THERMODYNAMICS OR BUSINESS ECONOMICS OR CONSTRUCTION BUILDING TECHNOLOGY)
AND **[excluding]: RESEARCH AREAS:** (PARASITOLOGY OR ARCHITECTURE OR PLANT SCIENCES OR MUSIC OR FISHERIES)

The search in CINAHL in 2021

1. physical activity
2. aerobic exercise
3. sport*
4. walking
5. physical training
6. 1 OR 2 OR 3 OR 4 OR 5
7. dementia
8. Alzheimer*
9. 7 OR 8
10. prospective study
11. longitudinal
12. follow-up
13. observational
14. cohort study
15. 10 OR 11 OR 12 OR 13 OR 14
16. 6 AND 9 AND 15

Filters: English, Human, All adult, 12/2017 – 10/2021, Research articles
Search field: Abstract

The search in Web of Science in 2021

1. ”physical activity”
2. ”aerobic exercise”
3. sport*
4. walking
5. ”physical training”
6. 1 OR 2 OR 3 OR 4 OR 5
7. dementia
8. Alzheimer*
9. 7 OR 8
10. prospective
11. longitudinal
12. follow-up
13. observational
14. cohort*
15. 10 OR 11 OR 12 OR 13 OR 14
16. 6 AND 9 AND 15

Articles (Document Types) and English (Languages) and 27TH ANNUAL MEETING OF THE SOCIETY FOR THE STUDY OF INGESTIVE BEHAVIOR SSIB or ALZHEIMER S ASSOCIATION INTERNATIONAL CONFERENCE (Exclude – Conference Titles) and Articles (Document Types) and Orthopedics or Surgery or Engineering or Medical Informatics or Obstetrics Gynecology or Urology Nephrology or Cell Biology or Dentistry Oral Surgery Medicine or Emergency Medicine or History or Mechanics or Oncology or Ophthalmology or Otorhinolaryngology or Respiratory System or Rheumatology or Social Work (Exclude – Research Areas)

Date range: 22.12.2017 – 21.10.2021

Field of search: Abstract

Specifications to the inclusion and exclusion criteria

Specifications to the inclusion and exclusion criteria at title and abstract screening phase in cases of disagreements:

The studies are included if

- The study is a longitudinal follow-up study addressing something else than physical activity as a dependent variable and dementia as an outcome but physical activity is mentioned as a covariate or it is unclear based on the abstract if physical activity is used as a covariate.
- The studies that have frailty as an independent variable with low physical activity is used as one of the criteria (or it is not clear what are the criteria according to the title and abstract) and dementia is the outcome variable.
- The studies that have healthy aging, survival or successful aging as an outcome measure with being undemented as one of the criteria or the criteria are not specified in the abstract and physical activity is one the dependent variables.

The studies are excluded if

- The study is a longitudinal follow-up study addressing something else than physical activity as a dependent variable and dementia as an outcome and covariates are listed in the abstract and physical activity is not one of them.
- Dementia mortality is the outcome variable.
- The study is considered to be cross-sectional because there is no indication in the title or the abstract that the study has a follow-up.
- The studies that are solely addressing the effects of scuba-diving or diving.
- The studies that are examining the effect of repetitive head trauma resulted from eg. boxing.
- The study addresses only a specific cohort of patients with a certain diagnosis or in for example “fallers”.
- Long-term physical activity during the follow-up is examined instead of baseline physical activity in only one time point.
- They study the effect of physical activity on structural brain changes and there is no indication in the title or abstract that dementia would also be measured.
- The study addresses the effect of sedentary hours in a day on cognition.
- The study addresses the effect of life-space mobility on dementia instead of the effect of physical activity.
- Baseline physical activity has not been measured or the estimate of physical activity is based on a single bout of physical activity.

Specifications to the precise inclusion and exclusion criteria at the full-text screening phase in cases of disagreements:

The study is included if

- Physical activity is only included as an independent variable that is a composite variable (eg. frailty) and the results for the components are mentioned separately, even if the actual numbers are not specified. If the results for the components are mentioned separately but no numbers are provided, the authors are contacted and asked for the numbers.
- There is a valid measure of baseline cognition or the population is in midlife. We define midlife as being 55 years old or younger and require that the cohort members are aged 55 years or less (mean age or median < 55 years and maximum age 65 years or +1 SD < 60 years).

The study is excluded if

- The study has used only orientation to date, day of week, month and year as a baseline measure of cognition.
- Studies in which dementia diagnosis was self-reported, based solely on disability scale or the main source (≥ 50%) of dementia diagnoses were death registers.

A quality assessment tool for the quality assessment of cohort studies addressing the association of physical activity and dementia or cognition

Note: A study can be given a maximum of one star for each numbered item within the Selection, Comparability and Outcome categories.

Selection

1) Representativeness of the exposed cohort

a) Truly representative (represents well the whole age group or the whole age group of any particular race and is not selected in regards to some disease, socio-economic status or for example only inhabitants of a nursing home, participation rate > 70% (of those alive) and sample size at least 1000 ***(one star)***

b) Truly or somewhat representative (participation rate 50-70%) and sample size > 1000 ***(half a star)***

c) Selected group in regards to some characteristic or participation rate < 50% or sample size < 1000

d) No description of the derivation of the cohort or participation rate lacks

2) Performance quality (adapted from (Br J Sports Med 2017;51:1410-18))

a) Good: PA assessed with a structured questionnaire of the duration, frequency and intensity of PA or the intensity of PA assessed with a structured question. Or PA assessed with an objective measure of PA (eg. accelerometer) **(one star)**

b) Moderate: Participation only in some types of sports assessed but other activities not considered or assessment of intensity lacks. Frequency or duration are assessed. **(half a star)**

c) Low: A “yes” or “no” question used. Frequency and duration not assessed. Or physical activity index on versatility of sports and somewhat physical household chores but not assessing intensity, frequency or duration. Or not described how exercise or physical activity was measured. (no star)

3) Demonstration that outcome of interest was not present at start of study

a) Yes. In a study population whose average age > 55 years, valid measure of cognition is used and demented individuals and individuals with mild cognitive impairment at baseline according to baseline cognition screening have been excluded or population is in midlife (mean age or median < 55 years and maximum age 65 years or +1 SD < 60 years) ***(one star)***

b) No

Comparability

1) Comparability of cohorts on the basis of the design or analysis controlled for confounders

a) The study controls for the following four factors: age, sex (or all cohort members represent the same sex), some vascular risk factor† and education or a measure of general cognitive ability at baseline (education criterion is not needed if all cohort

members have the same education level). In addition, the results have been adjusted with baseline cognition in study population whose average age > 55 years **(one star)**

b) The study controls only for three of the factors presented above (age, sex, some vascular risk factor and education or a measure of general intelligence) or/and in study populations whose mean age > 55 years the results have not been adjusted with baseline cognition or the sociodemographic and health behaviors controlled for are not specified further **(half a star)**

c) Cohorts are not comparable on the basis of covariates controlled for (no star)

Outcome
1) Assessment of outcome

a) A validated measure of dementia ***(one star)***

b) Record linkage ***(half a star)***

c) Self report or other

d) No description

2) Was follow-up long enough for outcomes to occur

a) Yes (***one star)***

b) No

Indicate the median duration of follow-up and a brief rationale for the assessment above: 10 years in dementia studies.

3) Adequacy of follow-up of cohorts

a) Complete follow up- all subject accounted for ***(one star)***

b) Subjects lost to follow up unlikely to introduce bias- number lost less than or equal to 20%* (when follow-up less than 10 years) or 30%* (when follow-up at least 10 years) of those alive or description of those lost suggested no different from those followed. ***(one star)***

c) Follow up rate less than 80%* (when follow-up less than 10 years) or less than 70%* (when follow-up longer than 10 years) and no description of those lost.

d) No statement

* Follow-up rates are calculated taking into account only the cohort members who have been alive at the time of the follow-up
† Vascular risk factor signifies a cardiovascular disease or information on smoking, body mass index, cholesterol levels, diet, blood pressure, diabetes or blood glucose.

Thresholds for converting the Newcastle-Ottawa scales to AHRQ standards (good, moderate, and poor):
Good quality: Selection: 2,5 - 3 stars, Comparability: 1 star, Outcome: 2,5 - 3 stars
Moderate quality: Selection: 2-3 stars, Comparability: 0,5 -1 star, Outcome: 2-3 stars,
Poor quality: studies not reaching Moderate/Good quality

A quality assessment tool for the quality assessment of case-control studies addressing the association of physical activity and dementia or cognition

Note: A study can be given a maximum of one star for each numbered item within the Selection, Comparability and Outcome categories.

Baseline

1) Selection of controls:

a) Community controls **(half a star)**

b) Hospital controls

c) No description

2) Definition of controls:

a) No history of disease (endpoint) **(half a star)**

b) No description of source

3) Demonstration that outcome of interest was not present at start of study

a) Yes. In a study population whose average age > 55 years, valid measure of cognition is used and demented individuals and individuals with mild cognitive impairment at baseline according to baseline cognition screening have been excluded or population is in midlife (mean age or median < 55 years and maximum age 65 years or +1 SD < 60 years) **(one star)**

b) No

4) Performance quality (adapted from (Br J Sports Med 2017;51:1410-18))

a) Good: PA assessed with a structured questionnaire of the duration, frequency and intensity of PA or the intensity of PA assessed with a structured question. Or PA assessed with an objective measure of PA (eg. accelerometer). Additionally same method is used for cases and controls and non-response rate is the same for cases and controls. **(one star)**

b) Moderate: Participation only in some types of sports assessed but other activities not considered or assessment of intensity lacks. Frequency or duration are assessed. **(half a star)**

c) Low: A “yes” or “no” question used. Frequency and duration not assessed. Or physical activity index on versatility of sports and somewhat physical household chores but not assessing intensity, frequency or duration. (no star)

Comparability

1) Comparability of cohorts on the basis of the design or analysis controlled for confounders

a) The study controls for the following four factors: age, sex (or all cohort members represent same sex), some vascular risk factor† and education or a measure of general cognitive ability at baseline. In addition, the results have been adjusted with baseline cognition in study population whose average age > 55 years **(one star)**

b) The study controls only for three of the factors presented above (age, sex, some vascular risk factor and education or a measure of general intelligence) or in study populations whose mean age > 55 years the results have not been adjusted with baseline cognition **(half a star)**

c) Cohorts are not comparable on the basis of covariates controlled for (no star)

Outcome

1) Assessment of outcome

a) A validated measure of dementia (if many cognitive tests used, most validated) ***(one star)***

b) Record linkage ***(half a star)***

c) Self report or other

d) No description

2) Was follow-up long enough for outcomes to occur

a) Yes (***one star)***

b) No

Indicate the median duration of follow-up and a brief rationale for the assessment above: 10 years in dementia studies.

3) Is the case definition adequate?:

a) Yes, with a valid measure of dementia **(half a star)**
b) No, does not fulfil the criteria defined above

4) Representativeness of the cases:

a) Consecutive or obviously representative series of cases **(half a star)**
b) Potential for selection biases or not stated

† Vascular risk factor signifies a cardiovascular disease or information on smoking, body mass index, cholesterol levels, diet, blood pressure, diabetes or blood glucose.

Thresholds for converting the Newcastle-Ottawa scales to AHRQ standards (good, moderate, and poor):
Good quality: Baseline: 2,5 - 3 stars, Comparability: 1 star, Outcome: 2,5 - 3 stars
Moderate quality: Baseline: 2-3 stars, Comparability: 0,5 -1 star, Outcome: 2-3 stars,
Poor quality: studies not reaching Moderate/Good quality

Deviations from the original study plan

1. We report the results in two parts
   - The numbers of subanalyses grew so large that we decided to split the systematic review and meta-analysis into two parts for clarity (one part for dementia and one part for cognition).
2. Our aim was to study what kind of physical activity is associated with decreased incidence of dementia
   - One aim was to compare the effect of the volume of physical activity and the intensity of physical activity but there weren't enough studies reporting a relation between the intensity of physical activity and dementia. Another aim was to examine the separately the effect of a rough measure of physical activity (yes or no question, number of different physical activities) or walking distance to more specific measures of physical activity (taking into account the volume, intensity and frequency of physical activity). During the examination of the physical activity measures of the included studies, we decided that it would be better and more clear way to categorize them by WHO physical activity recommendation threshold. This way, we can pinpoint a specific volume of physical activity and take into account whether the study actually is able to measure physical activity reliably enough to tell whether physical activity WHO recommendation is met. At this point, we also glanced the earlier meta-analyses more thoroughly and decided to do an analysis similar as in the most earlier meta-analyses (highest physical activity group vs lowest physical activity group) for better comparison with the earlier studies.
3. In addition to dementia and Alzheimer's disease, we collected the results separately for vascular dementia.
4. The method of measuring education was extracted with precision but was not examined with a separate sub-analysis because of vast heterogeneity in the classifications.
5. Data extraction of other modifiers than the length of the follow-up was done only by one researcher due to time and resource constraints.
6. The quality assessment tool was developed only after research plan was ready and adjusting for chronic diseases at baseline was not included because adjusting for education and some vascular risk factor was deemed more important.
7. We did not use Review Manager 5 but Covidence for handling data.
8. We conducted the prespecified moderator analyses but the classifications varied a little according to the data found (eg. follow-up length was grouped in a different way because we found many studies with follow-up length over 20 years).
   - Separate analyses were not done for according to the validity of physical activity measurement because we did a separate analysis according to meeting physical activity WHO recommendations as discussed earlier in point 2.
   - We did not perform separate analyses for different genders because there was not enough data: almost all studies presented results only for men and women jointly and very few studies reported the results for gender interaction test.
   - There was not enough data for a separate analysis for twin studies.
9. We aimed to include studies with "a valid physical activity questionnaire", but this criterion would have tremendously restricted the number of included studies, we ended up including also physical activity questionnaires that are not separately validated.

Supplementary analyses

Supplementary Table S1. Physical activity and all-cause dementia, supplementary analyses

Subanalyses	Pooled RR	95% Confidence interval	I^2	Number of studies combined	b estimate†	95% Confidence intervals
Prospective cohort studies [8, 10, 26-30, 33, 36-39, 41-62, 68-79]	0.81	0.77, 0.85	68.4%	46		
Case-control studies [34, 35, 40]	0.70	0.52, 0.95	73.4%	3		
All physical activity without the study with the largest weight (Palta et al. 2019)	0.80	0.77, 0.84	68.0%	48		
All physical activity without the study with the largest sample size (Kivimäki et al. 2019)	0.80	0.76, 0.84	70.1%	48		
Work-related physical activity [13, 69]	1.25	0.98, 1.59	29.4%	2		
**All PA**						
Moderators						
Sample size					1.00	1.00, 1.00
Baseline cognition *, adjusted	0.79	0.72, 0.88	54.3%	12		
not adjusted	0.81	0.77, 0.85	70.1%	37		
Education *, adjusted	0.80	0.76, 0.85	69.0%	38		
not adjusted	0.78	0.69, 0.88	64.4%	11		
Chronic diseases *, adjusted	0.80	0.75, 0.85	71.0%	28		
not adjusted	0.81	0.76, 0.87	56.1%	21		
APOE ε4 status *, adjusted	0.82	0.76, 0.88	56.6%	15		
not adjusted	0.79	0.75, 0.84	69.5%	34		
Funding source *, only non-commercial	0.79	0.75, 0.84	72.2%	41		
also commercial or not told	0.83	0.78, 0.89	4.1%	9		
Number of confounders					1.01	0.95, 1.08

* Moderate heterogeneity observed (I^2 up to 70.1%) in one or more subgroups; tests for heterogeneity between subgroups are likely to be invalid

† Beta estimate is the regression coefficient from the meta-regression examining the relationship of modifier or continuous physical activity on the log risk ratio of dementia.

Supplementary Figure S1. Longitudinal observational studies of physical activity and Alzheimer's disease: forest plot

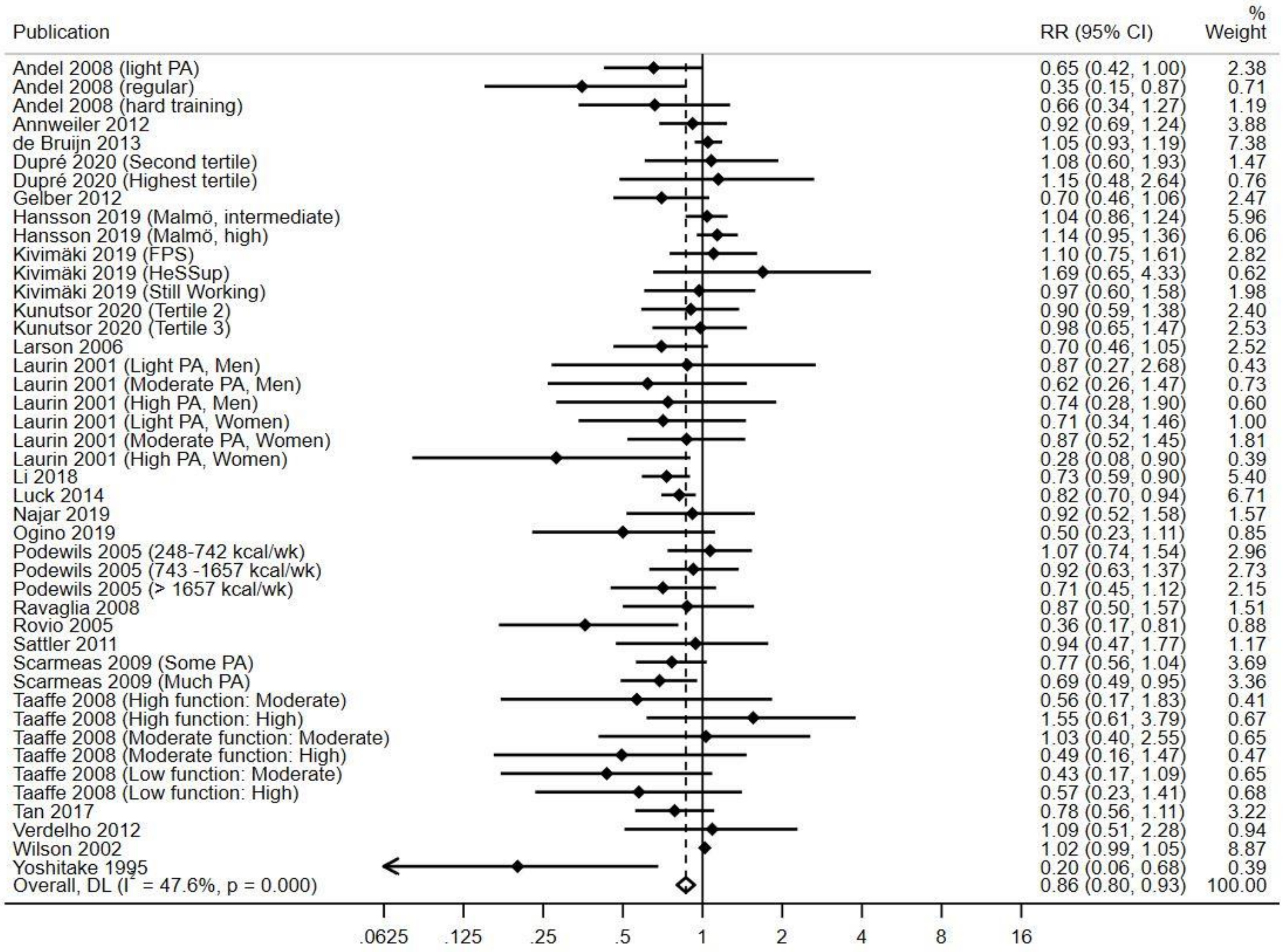

Supplementary Figure S2. Funnel plot for the longitudinal observational studies on physical activity and Alzheimer's disease with pseudo 95% confidence intervals.

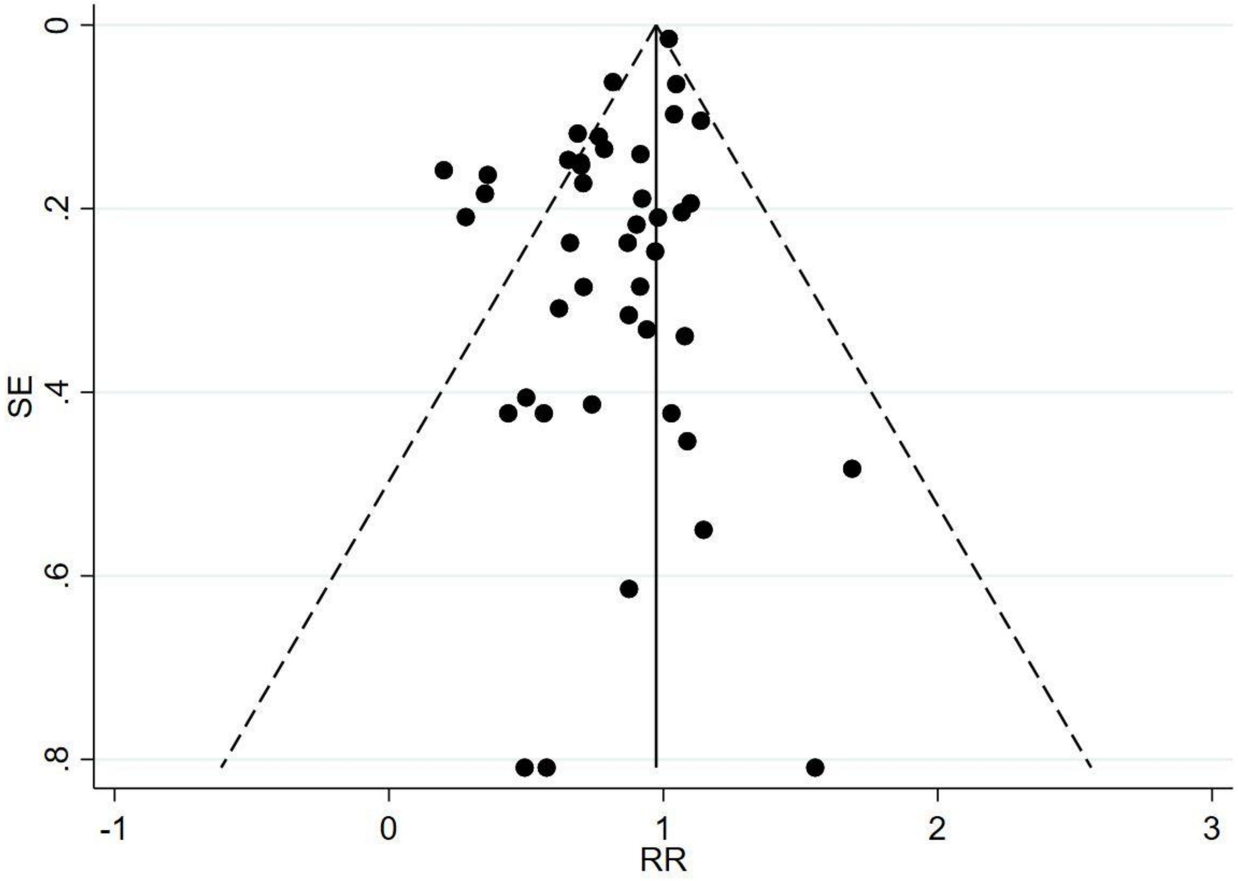

Supplementary Table S2. Physical activity and Alzheimer's disease, supplementary analyses

Subanalyses	Pooled RR	95% Confidence interval	I^2	Number of studies combined	b estimate†	95% Confidence intervals
Only leisure-time PA & quality moderate or high [11, 34, 77]	0.68	0.50, 0.92	45.2%	3		
Highest PA group vs lowest (1 highest vs 1 lowest)[8, 10, 11, 26, 29, 34, 37, 38, 40, 46, 47, 51, 56, 57, 60, 63, 65-68, 74, 77, 81, 82]	0.87	0.79, 0.95	55.7%	24		
Prospective cohort studies [8, 10, 11, 26, 29, 37, 38, 46, 47, 51, 56, 57, 60, 63, 65-68, 74, 77, 81, 82]	0.89	0.83, 0.96	43.3%	22		
Case-control studies [34, 40]	0.64	0.49, 0.83	0.0%	2		
All physical activity without the study with the largest weight (Hansson et al. 2019)	0.83	0.77, 0.91	48.5 %	23		
All physical activity without the study with the largest sample size (Kivimäki et al. 2019)	0.85	0.78, 0.92	50.4%	23		
**All PA**						
Moderators						
Sample size					1.00	1.00, 1.00
Baseline cognition †, adjusted	0.84	0.74, 0.95	41.6%	9		
not adjusted	0.88	0.80, 0.97	44.1%	15		
Education †, adjusted	0.85	0.78, 0.92	36.1%	20		
not adjusted	0.92	0.73, 1.16	45.9%	4		
Chronic diseases †, adjusted	0.82	0.74, 0.91	43.6%	17		
not adjusted	0.95	0.85, 1.06	26.7%	7		
APOE ε4 status ‡, adjusted	0.85	0.76, 0.94	53.5%	13		
not adjusted	0.88	0.77, 0.99	37.9%	11		
Funding source ‡, only non-commercial	0.86	0.79, 0.93	55.9%	22		
also commercial or not told	0.92	0.75, 1.12	0.0%	3		
Number of confounders					1.00	0.90, 1.12

* Beta estimate is the regression coefficient from the meta-regression examining the relationship of modifier or continuous physical activity on the log risk ratio of dementia

† Heterogeneity for between groups non-significant (P=.539 for baseline cognition, P=.489 for education, P=.059 for chronic diseases)

‡ Moderate heterogeneity observed (I^2 up to 70.1%) in one or more subgroups; tests for heterogeneity between subgroups are likely to be invalid

Supplementary Figure S3. Longitudinal observational studies of physical activity and vascular dementia: forest plot

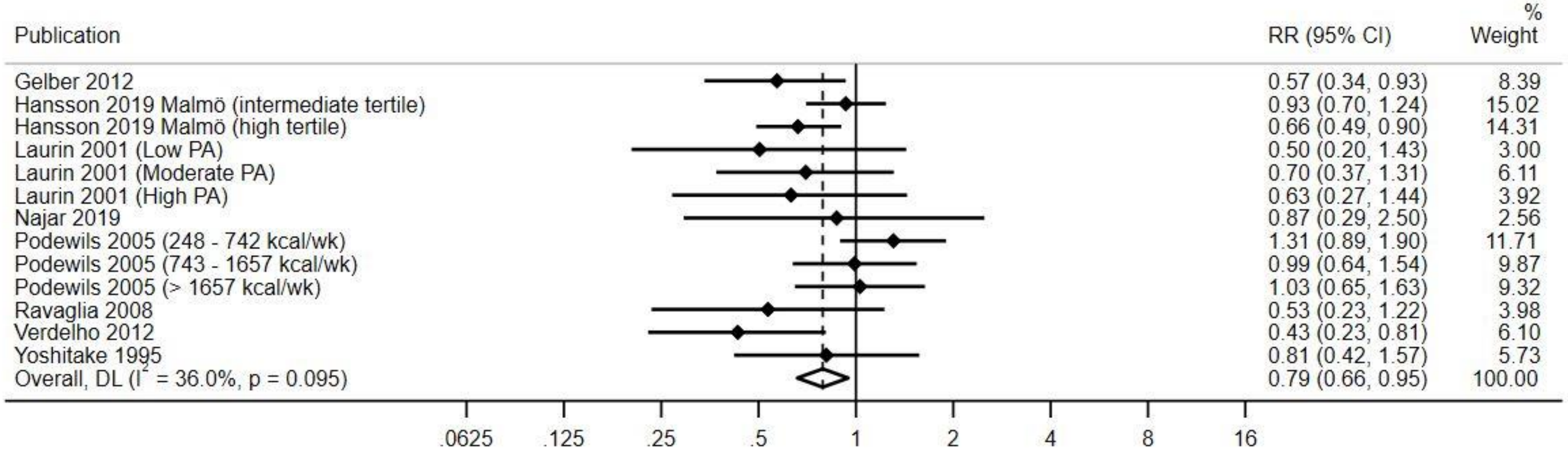

Supplementary Figure S4. Funnel plot for the longitudinal observational studies on physical activity and vascular dementia with pseudo 95% confidence intervals.

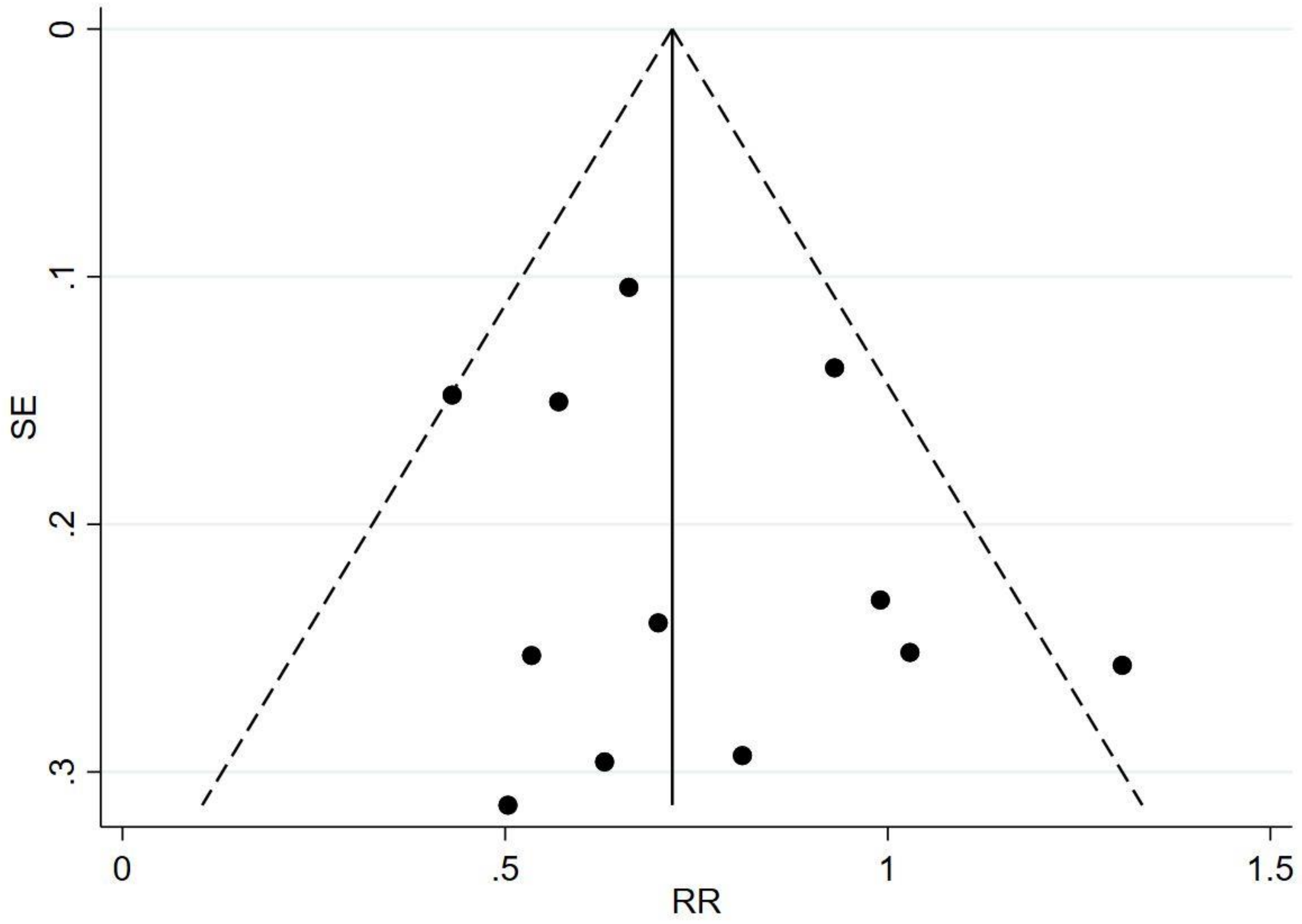

Supplementary Table S3. PA and VaD: main results, main sensitivity analyses with meta-regressions and subgroup analyses, and dose-response analysis

	Pooled RR	95% Confidence interval	I^2	Number of cohorts combined	beta estimate ‡	95% Confidence interval
**All PA**	0.79	0.66, 0.95	36.0%	8		
Baseline age (continuous)					1.00	0.92, 1.10
Baseline age (categorical						
Age group 30-65 years [28, 42, 75]	0.75	0.59, 0.95	26.4%	3		
Age group 70-85 years [11, 49, 53, 62, 69]	0.81	0.62, 1.04	41.3%	5		
Follow-up length					1.00	0.89, 1.13
Follow-up length < 10 years [11, 49, 53, 62, 69]	0.81	0.62, 1.04	41.3%	5		
Follow-up length ≥10 years [28, 42, 75]	0.75	0.59, 0.95	26.4%	3		
Study quality (low vs moderate vs high)*					1.26	0.38, 4.13
Low quality [11, 28, 62, 69]	0.87	0.68, 1.10	55.3%	4		
Moderate quality [49, 53, 75]	0.64	0.44, 0.92	0.0%	3		
High quality † [42]	0.57	0.34, 0.94	0.0%	1		
Meeting PA guidelines [11, 28, 49, 53, 62, 69]	0.74	0.59, 0.93	22.7%	6		
Not meeting PA guidelines [11, 28, 49, 75]	0.95	0.73, 1.23	23.9%	4		
Age group 30-55 years, Quality high & Follow-up length > 20 years † [42]	0.57	0.34, 0.94	0.0%	1		

Abbreviations: RR= Relative risk, I^2=Heterogeneity, PA=Physical activity, VaD=Vascular dementia

* Study quality was assessed with a quality assessment tool we developed (See Supplementary Material Part 1 for details).

† Not a meta-analytical analysis, only one study

‡ Beta estimate is the regression coefficient from the meta-regression examining the relationship of modifier or continuous PA on the log risk ratio of dementia.

§ The test for heterogeneity between groups was non-significant (P=.159).

Supplementary Table S4. Physical activity and vascular dementia, supplementary analyses

Subanalyses	Pooled RR	95% Confidence interval	I^2	Number of studies combined	b estimate‡	95% Confidence intervals
Only leisure-time PA & quality moderate or high*	-	-	-	-		
Highest PA group vs lowest (1 highest vs 1 lowest) [10, 26, 40, 47, 51, 60, 67, 73]	0.68	0.56, 0.82	0.0%	8		
Prospective cohort studies [10, 26, 47, 51, 60, 67, 73]	0.81	0.68, 0.98	34.0%	7		
Case-control studies † [40]	0.57	0.34, 0.93	0.0%	1		
All physical activity without the study with the largest weight (Hansson et al. 2019)	0.78	0.62, 0.98	37.7%	7		
All physical activity without the study with the largest sample size (Hansson et al. 2019)	0.78	0.62, 0.98	37.7%	7		
**All PA**						
Moderators						
Sample size					1.00	1.00, 1.00
Baseline cognition §, adjusted	0.91	0.65, 1.27	55.9%	3		
not adjusted	0.73	0.62, 0.86	0.0%	5		
Education §, adjusted	0.78	0.64, 0.96	46.6%	6		
not adjusted	0.83	0.47, 1.45	0.0%	2		
Chronic diseases §, adjusted	0.73	0.62, 0.87	0.0%	6		
not adjusted	0.92	0.62, 1.37	65.7%	2		
APOE ε4 status §, adjusted	0.90	0.65, 1.24	54.0%	3		
not adjusted	0.74	0.62, 0.87	0.0%	5		
Funding source, only non-commercial	0.81	0.66, 1.00	47.5%	7		
also commercial or not told †	0.63	0.40, 0.99	0.0%	1		
Number of confounders					1.03	0.85, 1.26

* No studies

† Not meta-analytical analysis, only one study

‡ Beta estimate is the regression coefficient from the metaregression examining the relationship of modifier or continuous physical activity on the log risk ratio of dementia.

§ The test for heterogeneity between groups was non-significant (P=.248 for baseline cognition, P=.846 for education, P=.305 for chronic diseases, P=.294 for apolipoprotein E ε4 allele).

Supplementary Figure S5. Physical activity and all-cause dementia, Alzheimer's disease and vascular dementia in APOE ε4 carriers

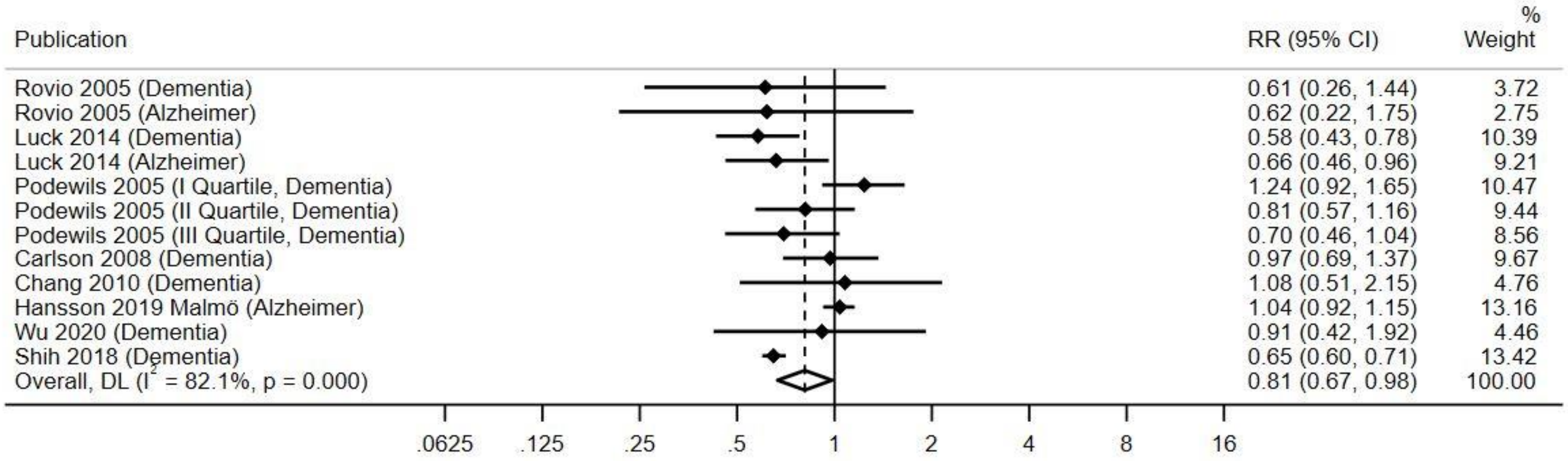

BMJ Publishing Group Limited (BMJ) disclaims all liability and responsibility arising from any reliance placed on this supplemental material which has been supplied by the author(s)

Supplementary Figure S6. Physical activity and all-cause dementia, Alzheimer's disease and vascular dementia in APOE ε4 carriers when study quality is good or moderate

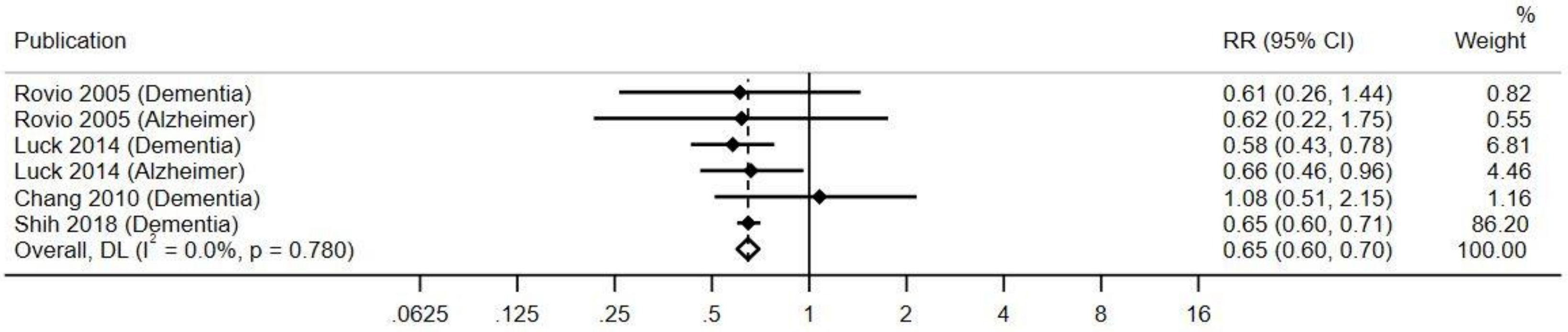

Supplementary Figure S7. Physical activity and all-cause dementia, Alzheimer's disease and vascular dementia in APOE ε4 non-carriers

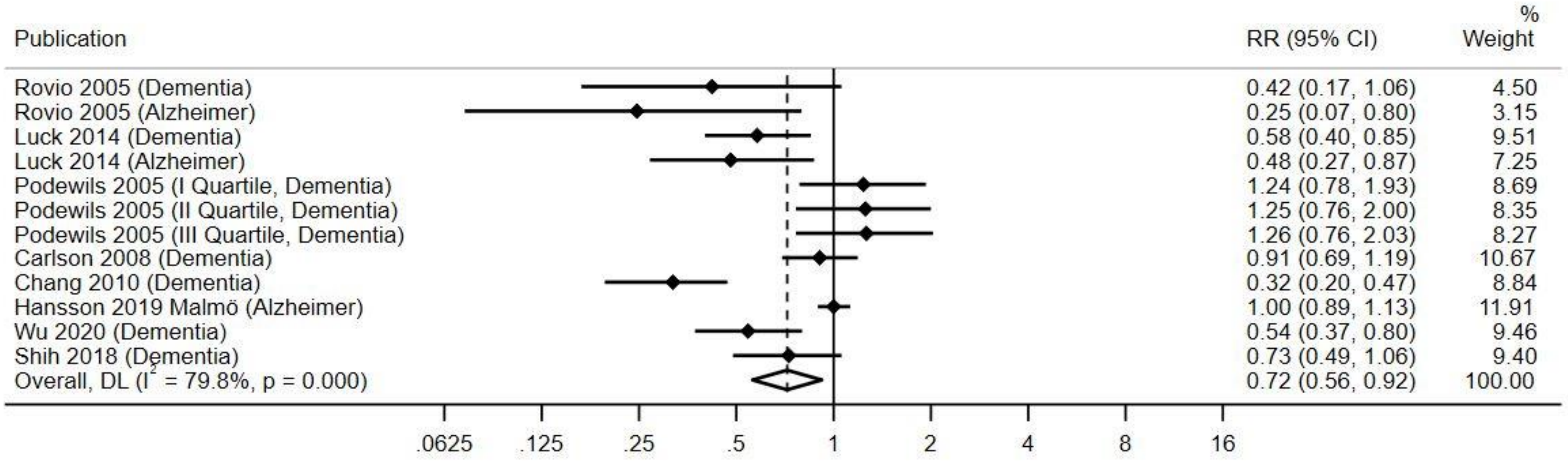

Supplementary Figure S8. Physical activity and all-cause dementia, Alzheimer's disease and vascular dementia in APOE ε4 non-carriers when study quality is good or moderate

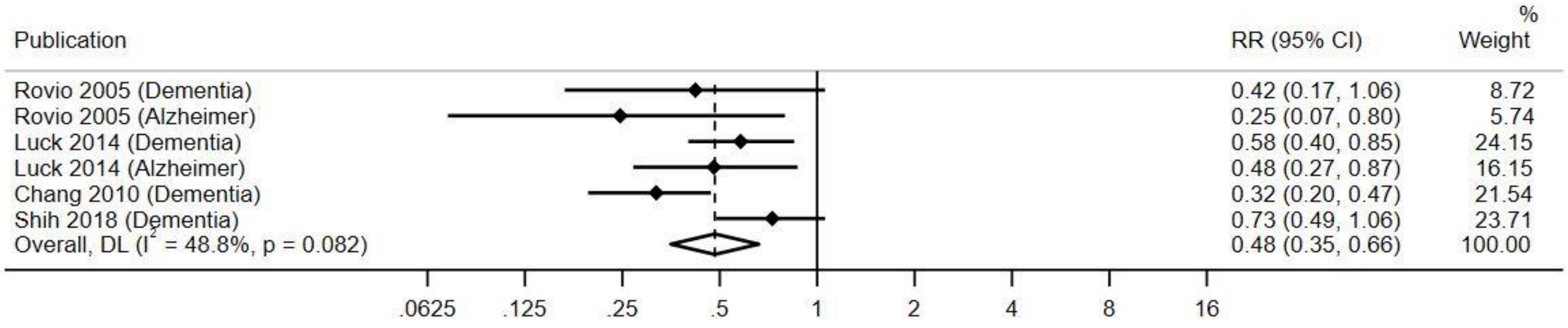

Sensitivity analysis for APOE ε4 allele

To find out if APOE ε4 allele moderates the association between physical activity and dementia, we gathered information from the individual studies on APOE ε4 allele interaction analysis results and results reported separately for APOE ε4 carriers and noncarriers. 12 studies presented results from APOE ε4 interaction analyses (Hansson et al. 2019, Dupré et al. 2020, Tan et al. 2017, Taaffe et al. 2008, Podewils et al. 2005, Kim et al. 2011, Ravaglia et al. 2008, Lindsay et al. 2002, Paillard-Borg et al. 2009, Luck et al. 2014, Rovio et al. 2005, Shih et al. 2018). Most studies reported only results from interaction test not separating multiplicative and additive interaction, one study reported additive but not multiplicative interaction results and two studies reported both multiplicative and additive interaction results. The results were the following:

- 12 studies reported interaction analysis results
- 9 studies reported no interaction ((Hansson et al. 2019, Dupré et al. 2020, Tan et al. 2017, Taaffe et al. 2008, Rovio et al. 2005, Podewils et al. 2005, Luck et al. 2014, Kim et al. 2011, Ravaglia et al. 2008, Lindsay et al. 2002, Paillard-Borg et al. 2009)
- 2 studies reported no multiplicative interaction but significant or probable additive interaction (Luck et al. 2014, Rovio et al. 2005)
- 1 study reported no interaction on additive scale. The results were presented stratified according to APOE ε4 status but the result for multiplicative interaction was not given (Shih 2018).
