# Supplementary material for: Physical activity as a protective factor for dementia and Alzheimer’s disease: systematic review, meta-analysis and quality assessment of cohort and case–control studies
Source: Br J Sports Med. 2022 Mar 17;56(12):701–9. doi: 10.1136/bjsports-2021-104981 (PMC9163715; doi:10.1136/bjsports-2021-104981)
Supplement: Supplementary data [file bjsports-2021-104981supp003.pdf]

Supplementary Material Part 3
Dose-response meta-analyses

Contents

Method description 1
Results - All-cause dementia (A-CD) 2
Linear model including all studies (A-CD) 2
Models including studies with 3 or more PA exposure levels (A-CD) 4
Results - Alzheimer's disease (AD) 7
Linear model including all studies (AD) 7
Models including studies with 3 or more PA exposure levels (AD) 9
Results - Vascular dementia (VD) 13
Linear model including all studies (VD) 13
Models including studies with 3 or more PA exposure levels (VD) 15
References 19

Method description

Calculation of PA exposure levels

Following the procedure of Blond et al (1), we used the midpoint of the physical activity range (mean or median) from each group as the value for PA exposure. MET values for listed activities were taken from the included articles, or estimated using MET values of 3.5 for walking, 4.5 for moderate physical activity and 8.0 for sports participation or vigorous PA. For studies in which PA was assessed as bouts per week, one bout was estimated to be 30 minutes in duration unless otherwise specified in the included article. When physical activity levels were specified in calories per week, mean body weights reported in the articles were used for calculating MET-minutes using the formula

$$MET * minutes = \frac{60 * kcal}{kg}$$

Where mean body weight was not reported in an article, continental body weight averages were used in the calculation (2). In studies where it was not possible to directly calculate MET*minutes per week for each group, we imputed PA exposure values using the means from other similar studies. We used a cutoff of a maximum of 21 hours of moderate PA per week (3 hours X 7 days), and this corresponds to a maximum value of 5040 MET-minutes per week.

Dose-response meta-analyses

Dose-response meta-analyses were performed using the dosresmeta package (3) in R and visualizations were created using the shiny webapp based on this package (4). Two-stage random effects meta-analyses using the restricted maximum likelihood procedure were used to pool RRs. Among studies with at least 3 different PA exposure levels, dose–response meta-analyses explored linear, quadratic and restricted cubic spline trends within the data. Knots in the restricted cubic spline regression models were set at the 20th and 80th percentiles of the overall PA exposure distribution. As it was not possible to explore quadratic or spline trends among studies with only two different PA exposure levels, we only examined linear trends

within this larger dataset that included all studies with at least two discrete PA exposure groups. We chose 200 MET min/week as the reference for the dose–response analyses as this was roughly equivalent to the mean MET*min per week value of reference groups in the included studies. Post-estimations based on the dose–response model were conducted to predict RRs and 95% CIs at specific MET*min per week values (approximately 200, 900, 2000, 3000, 4000, and 5000).

Results - All-cause dementia (A-CD)

Linear model including all studies (A-CD)

Scatter plot of RRs vs original exposure variable

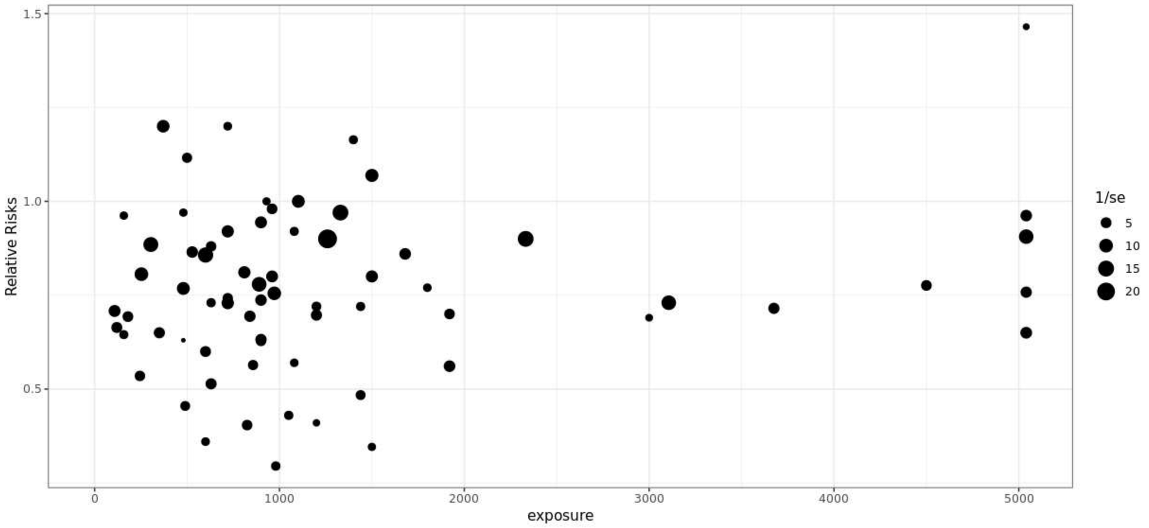

Linear Trend

```
Call:  dosresmeta(formula = logrr ~ exposure, id = id, type = type,
    cases = cases, n = n, data = dataset(), se = se, covariance = input$pscorr)

Two-stage random-effects meta-analysis
Estimation method: REML
Covariance approximation: Greenland & Longnecker

Chi2 model: X2 = 34.6275 (df = 1), p-value = 0.0000

Fixed-effects coefficients
             Estimate  Std. Error        z  Pr(>|z|)  95%ci.lb  95%ci.ub
(Intercept)   -0.0002      0.0000  -5.8845    0.0000   -0.0003   -0.0001  ***
---
Signif. codes:  0 ‘***’ 0.001 ‘**’ 0.01 ‘*’ 0.05 ‘.’ 0.1 ‘ ’ 1

Between-study random-effects (co)variance components
  Std. Dev
```

```
    0.0002

Univariate Cochran Q-test for residual heterogeneity:
Q = 123.3919 (df = 42), p-value = 0.0000
I-square statistic = 66.0%

43 studies, 43 values, 1 fixed and 1 random-effects parameters
   logLik        AIC        BIC
 276.7986  -549.5973  -546.1220
```

Graphical prediction

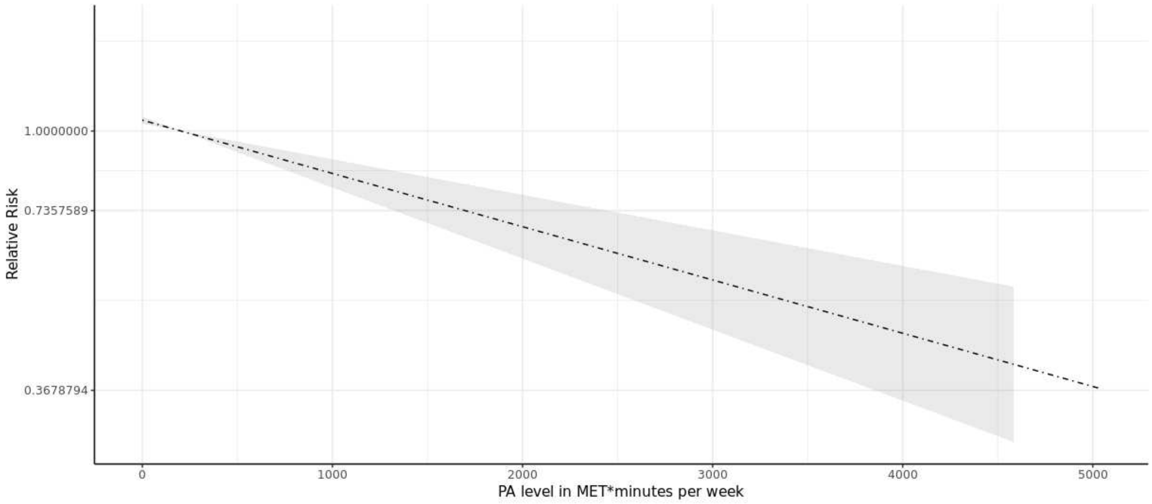

Analytical predictions

PA exposure	pred.lin	ci.lb.lin	ci.ub.lin
203.64	1.00	1.00	1.00
916.36	0.86	0.82	0.91
1934.55	0.70	0.62	0.79
2952.73	0.57	0.47	0.69
3970.91	0.46	0.36	0.60
4989.09	0.37	0.27	0.52

Models including studies with 3 or more PA exposure levels (A-CD)

Scatter plot of RRs vs original exposure variable

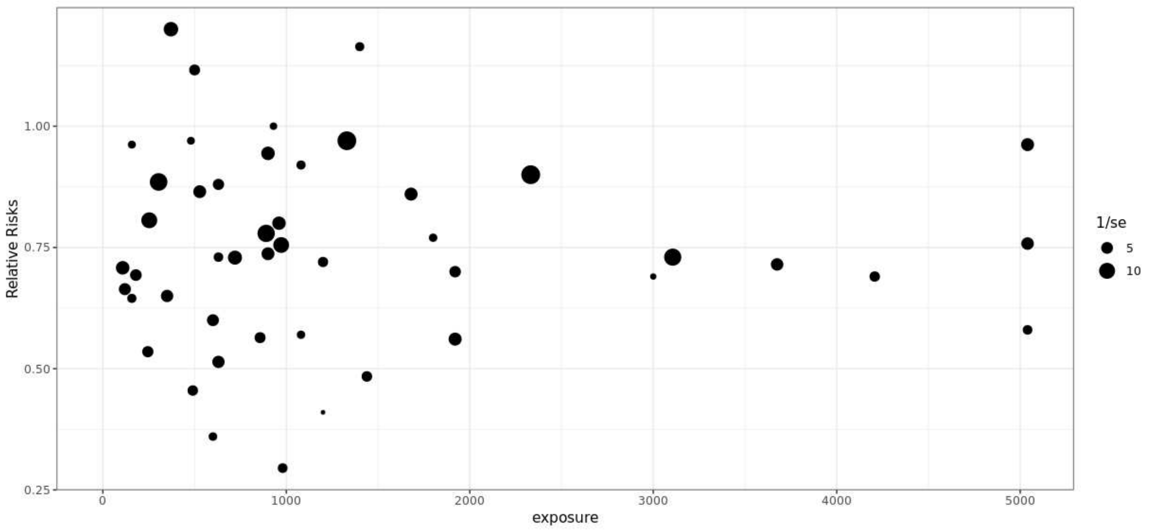

Linear Trend

```
Call:  dosresmeta(formula = logrr ~ exposure, id = id, type = type,
    cases = cases, n = n, data = dataset(), se = se, covariance = input$pscorr)

Two-stage random-effects meta-analysis
Estimation method: REML
Covariance approximation: Greenland & Longnecker

Chi2 model: X2 = 21.4096 (df = 1), p-value = 0.0000

Fixed-effects coefficients
             Estimate  Std. Error        z  Pr(>|z|)  95%ci.lb  95%ci.ub
(Intercept)   -0.0002      0.0000  -4.6270    0.0000   -0.0003   -0.0001  ***
---
Signif. codes:  0 '***' 0.001 '**' 0.01 '*' 0.05 '.' 0.1 ' ' 1

Between-study random-effects (co)variance components
  Std. Dev
    0.0001

Univariate Cochran Q-test for residual heterogeneity:
Q = 57.7815 (df = 19), p-value = 0.0000
I-square statistic = 67.1%
```

4

```
20 studies, 20 values, 1 fixed and 1 random-effects parameters
   logLik        AIC        BIC
 128.2688  -252.5375  -250.6487
```

Spline model

```
Call:  dosresmeta(formula = logrr ~ rcs(exposure, knots), id = id, type = type,
    cases = cases, n = n, data = dataset(), se = se, covariance = input$pscorr)

Two-stage random-effects meta-analysis
Estimation method: REML
Covariance approximation: Greenland & Longnecker

Chi2 model: X2 = 37.6403 (df = 2), p-value = 0.0000

Fixed-effects coefficients
                                           Estimate  Std. Error        z
rcs(exposure, knots)exposure.(Intercept)    -0.0005      0.0001  -5.5896
rcs(exposure, knots)exposure'.(Intercept)    0.0004      0.0001   4.5834
                                           Pr(>|z|)  95%ci.lb  95%ci.ub
rcs(exposure, knots)exposure.(Intercept)     0.0000   -0.0006   -0.0003  ***
rcs(exposure, knots)exposure'.(Intercept)    0.0000    0.0002    0.0006  ***
---
Signif. codes:  0 ‘***’ 0.001 ‘**’ 0.01 ‘*’ 0.05 ‘.’ 0.1 ‘ ’ 1

Between-study random-effects (co)variance components
                               Std. Dev                          Corr
rcs(exposure, knots)exposure     0.0002  rcs(exposure, knots)exposure
rcs(exposure, knots)exposure'    0.0001                            -1

Univariate Cochran Q-test for residual heterogeneity:
Q = 58.7637 (df = 38), p-value = 0.0169
I-square statistic = 35.3%

20 studies, 40 values, 2 fixed and 3 random-effects parameters
   logLik        AIC        BIC
 222.9501  -435.9001  -427.7122
```

Quadratic trend

```
Call:  dosresmeta(formula = logrr ~ exposure + I(exposure^2), id = id,
    type = type, cases = cases, n = n, data = dataset(), se = se,
    covariance = input$pscorr)

Two-stage random-effects meta-analysis
Estimation method: REML
Covariance approximation: Greenland & Longnecker

Chi2 model: X2 = 38.9658 (df = 2), p-value = 0.0000

Fixed-effects coefficients
                           Estimate  Std. Error        z  Pr(>|z|)  95%ci.lb
exposure.(Intercept)        -0.0004      0.0001  -4.6303    0.0000   -0.0005
I(exposure^2).(Intercept)    0.0000      0.0000   3.3398    0.0008    0.0000
                           95%ci.ub
exposure.(Intercept)        -0.0002  ***
I(exposure^2).(Intercept)    0.0000  ***
---
Signif. codes:  0 ‘***’ 0.001 ‘**’ 0.01 ‘*’ 0.05 ‘.’ 0.1 ‘ ’ 1

Between-study random-effects (co)variance components
               Std. Dev      Corr
exposure         0.0002  exposure
I(exposure^2)    0.0000        -1

Univariate Cochran Q-test for residual heterogeneity:
Q = 66.0653 (df = 38), p-value = 0.0032
I-square statistic = 42.5%

20 studies, 40 values, 2 fixed and 3 random-effects parameters
   logLik        AIC        BIC
 382.8104  -755.6209  -747.4329
```

Graphical prediction

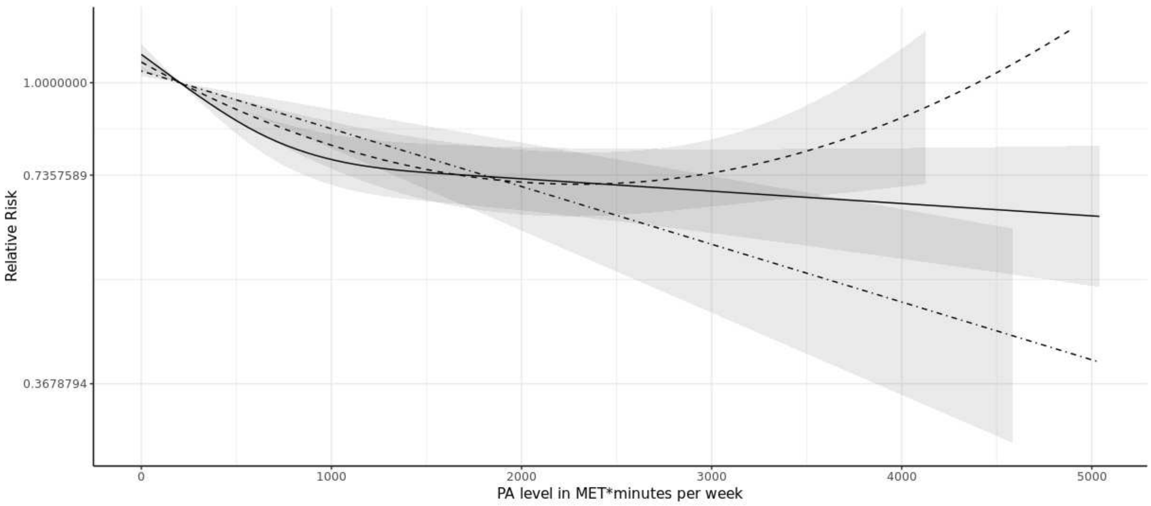

Linear Trend (dot-dash); Spline Model with knots at 20 and 80% of distribution (solid); Quadratic Trend (dashed)

Analytical predictions

exposure	pred.lin	ci.lb.lin	ci.ub.lin	pred.spl	ci.lb.spl	ci.ub.spl	pred.quadr	ci.lb.quadr	ci.ub.quadr
203.64	1.00	1.00	1.00	1.00	1.00	1.00	1.00	1.00	1.00
916.36	0.87	0.82	0.92	0.79	0.73	0.85	0.83	0.77	0.89
1934.55	0.72	0.62	0.83	0.73	0.66	0.81	0.72	0.65	0.80
2952.73	0.59	0.47	0.74	0.70	0.61	0.80	0.74	0.66	0.82
3970.91	0.49	0.36	0.66	0.67	0.56	0.80	0.88	0.71	1.11
4989.09	0.40	0.27	0.59	0.64	0.51	0.81	1.24	0.78	1.99

Results - Alzheimer’s disease (AD)

Linear model including all studies (AD)

Scatter plot of RRs vs original exposure variable

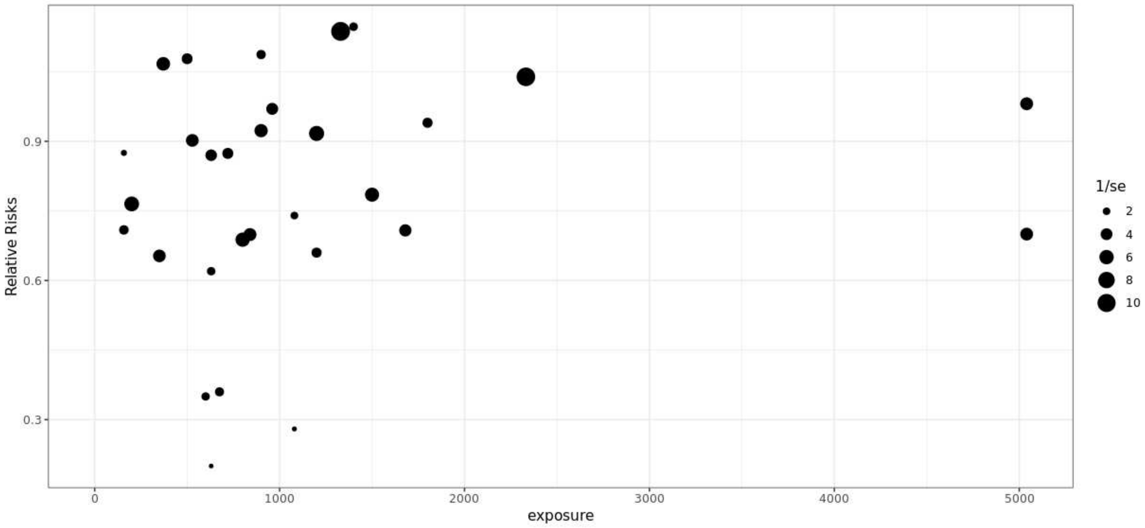

Linear Trend

```
Call:  dosresmeta(formula = logrr ~ exposure, id = id, type = type,
    cases = cases, n = n, data = dataset(), se = se, covariance = input$pscorr)

Two-stage random-effects meta-analysis
Estimation method: REML
Covariance approximation: Greenland & Longnecker

Chi2 model: X2 = 5.8708 (df = 1), p-value = 0.0154

Fixed-effects coefficients
             Estimate  Std. Error        z  Pr(>|z|)  95%ci.lb  95%ci.ub
(Intercept)   -0.0001      0.0000  -2.4230    0.0154   -0.0002   -0.0000  *
---
Signif. codes:  0 ‘***’ 0.001 ‘**’ 0.01 ‘*’ 0.05 ‘.’ 0.1 ‘ ’ 1

Between-study random-effects (co)variance components
  Std. Dev
    0.0001

Univariate Cochran Q-test for residual heterogeneity:
Q = 28.3221 (df = 18), p-value = 0.0573
I-square statistic = 36.4%

19 studies, 19 values, 1 fixed and 1 random-effects parameters
   logLik        AIC        BIC
```

8

```
118.7244  -233.4489  -231.6681
```

Graphical prediction

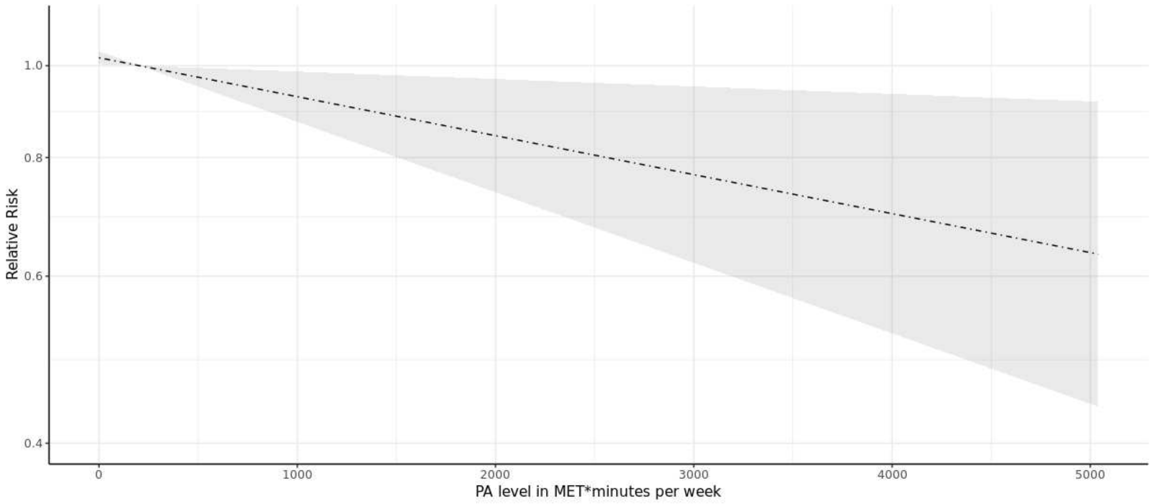

Analytical predictions

PA exposure	pred.lin	ci.lb.lin	ci.ub.lin
203.64	1.00	1.00	1.00
916.36	0.93	0.89	0.99
1934.55	0.85	0.74	0.97
2952.73	0.77	0.62	0.95
3970.91	0.70	0.52	0.93
4989.09	0.64	0.44	0.92

Models including studies with 3 or more PA exposure levels (AD)

Scatter plot of RRs vs original exposure variable

Iso-Markku P, *et al. Br J Sports Med* 2022; 56:701–709. doi: 10.1136/bjsports-2021-104981

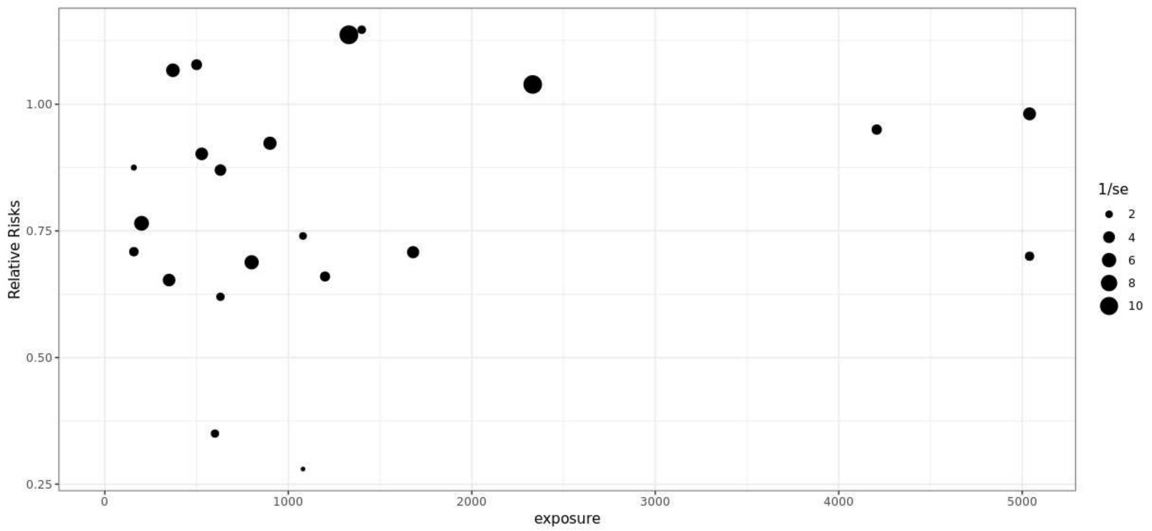

Linear Trend

```
Call:  dosresmeta(formula = logrr ~ exposure, id = id, type = type,
    cases = cases, n = n, data = dataset(), se = se, covariance = input$pscorr)

Two-stage random-effects meta-analysis
Estimation method: REML
Covariance approximation: Greenland & Longnecker

Chi2 model: X2 = 2.1500 (df = 1), p-value = 0.1426

Fixed-effects coefficients
             Estimate  Std. Error        z  Pr(>|z|)  95%ci.lb  95%ci.ub
(Intercept)   -0.0001      0.0001  -1.4663    0.1426   -0.0002    0.0000
---
Signif. codes:  0 ‘***’ 0.001 ‘**’ 0.01 ‘*’ 0.05 ‘.’ 0.1 ‘ ’ 1

Between-study random-effects (co)variance components
  Std. Dev
    0.0001

Univariate Cochran Q-test for residual heterogeneity:
Q = 10.8651 (df = 8), p-value = 0.2095
I-square statistic = 26.4%

9 studies, 9 values, 1 fixed and 1 random-effects parameters
   logLik        AIC        BIC
```

```
  55.4683  -106.9365  -106.7776
```

Spline model

```
Call:  dosresmeta(formula = logrr ~ rcs(exposure, knots), id = id, type = type,
    cases = cases, n = n, data = dataset(), se = se, covariance = input$pscorr)

Two-stage random-effects meta-analysis
Estimation method: REML
Covariance approximation: Greenland & Longnecker

Chi2 model: X2 = 6.2649 (df = 2), p-value = 0.0436

Fixed-effects coefficients
                                           Estimate  Std. Error        z
rcs(exposure, knots)exposure.(Intercept)    -0.0004      0.0001  -2.4593
rcs(exposure, knots)exposure'.(Intercept)    0.0003      0.0001   2.3245
                                           Pr(>|z|)  95%ci.lb  95%ci.ub
rcs(exposure, knots)exposure.(Intercept)     0.0139   -0.0007   -0.0001  *
rcs(exposure, knots)exposure'.(Intercept)    0.0201    0.0001    0.0006  *
---
Signif. codes:  0 ‘***’ 0.001 ‘**’ 0.01 ‘*’ 0.05 ‘.’ 0.1 ‘ ’ 1

Between-study random-effects (co)variance components
                               Std. Dev                          Corr
rcs(exposure, knots)exposure     0.0000  rcs(exposure, knots)exposure
rcs(exposure, knots)exposure'    0.0000                        0.9723

Univariate Cochran Q-test for residual heterogeneity:
Q = 16.9974 (df = 16), p-value = 0.3858
I-square statistic = 5.9%

9 studies, 18 values, 2 fixed and 3 random-effects parameters
   logLik        AIC        BIC
  85.2275  -160.4549  -156.5920
```

Quadratic trend

```
Call:  dosresmeta(formula = logrr ~ exposure + I(exposure^2), id = id,
    type = type, cases = cases, n = n, data = dataset(), se = se,
```

```
    covariance = input$pscorr)

Two-stage random-effects meta-analysis
Estimation method: REML
Covariance approximation: Greenland & Longnecker

Chi2 model: X2 = 2.8700 (df = 2), p-value = 0.2381

Fixed-effects coefficients
                           Estimate  Std. Error        z  Pr(>|z|)  95%ci.lb
exposure.(Intercept)        -0.0002      0.0001  -1.6085    0.1077   -0.0004
I(exposure^2).(Intercept)    0.0000      0.0000   1.3787    0.1680   -0.0000
                           95%ci.ub
exposure.(Intercept)         0.0000
I(exposure^2).(Intercept)    0.0000
---
Signif. codes:  0 ‘***’ 0.001 ‘**’ 0.01 ‘*’ 0.05 ‘.’ 0.1 ‘ ’ 1

Between-study random-effects (co)variance components
               Std. Dev      Corr
exposure         0.0002  exposure
I(exposure^2)    0.0000        -1

Univariate Cochran Q-test for residual heterogeneity:
Q = 19.6762 (df = 16), p-value = 0.2352
I-square statistic = 18.7%

9 studies, 18 values, 2 fixed and 3 random-effects parameters
   logLik        AIC        BIC
 158.5896  -307.1793  -303.3163
```

Graphical prediction

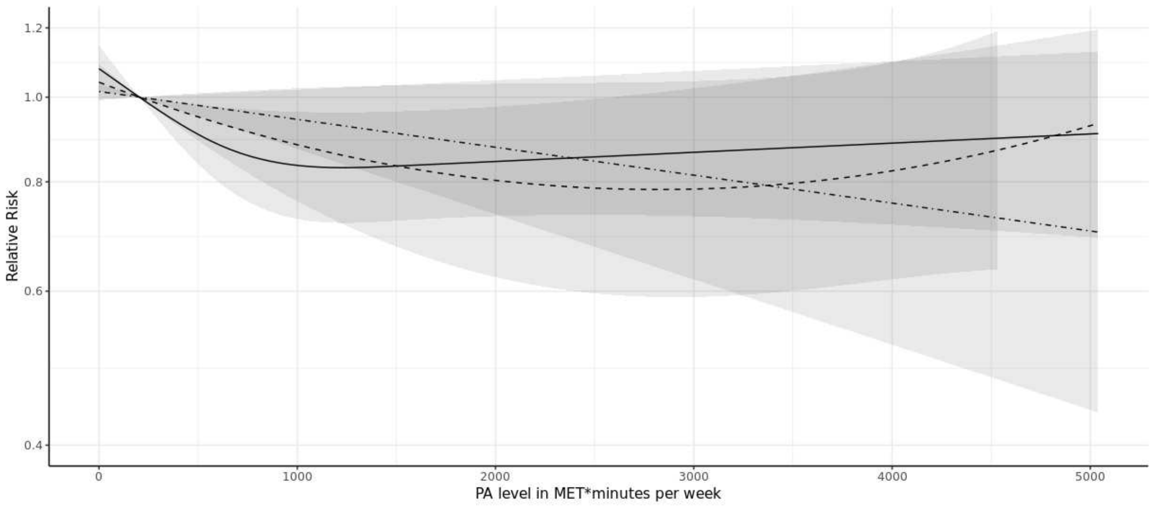

Linear Trend (dot-dash); Spline Model with knots at 20 and 80% of distribution (solid); Quadratic Trend (dashed)

Analytical predictions

PA exposure	pred.lin	ci.lb.lin	ci.ub.lin	pred.spl	ci.lb.spl	ci.ub.spl	pred.quadr	ci.lb.quadr	ci.ub.quadr
203.64	1.00	1.00	1.00	1.00	1.00	1.00	1.00	1.00	1.00
916.36	0.95	0.88	1.02	0.84	0.73	0.96	0.89	0.78	1.02
1934.55	0.88	0.74	1.04	0.84	0.73	0.97	0.81	0.63	1.04
2952.73	0.82	0.62	1.07	0.86	0.73	1.02	0.78	0.59	1.04
3970.91	0.76	0.52	1.10	0.89	0.72	1.10	0.82	0.62	1.09
4989.09	0.70	0.44	1.13	0.91	0.69	1.19	0.93	0.64	1.34

Results - Vascular dementia (VD)

Linear model including all studies (VD)

Scatter plot of RRs vs original exposure variable

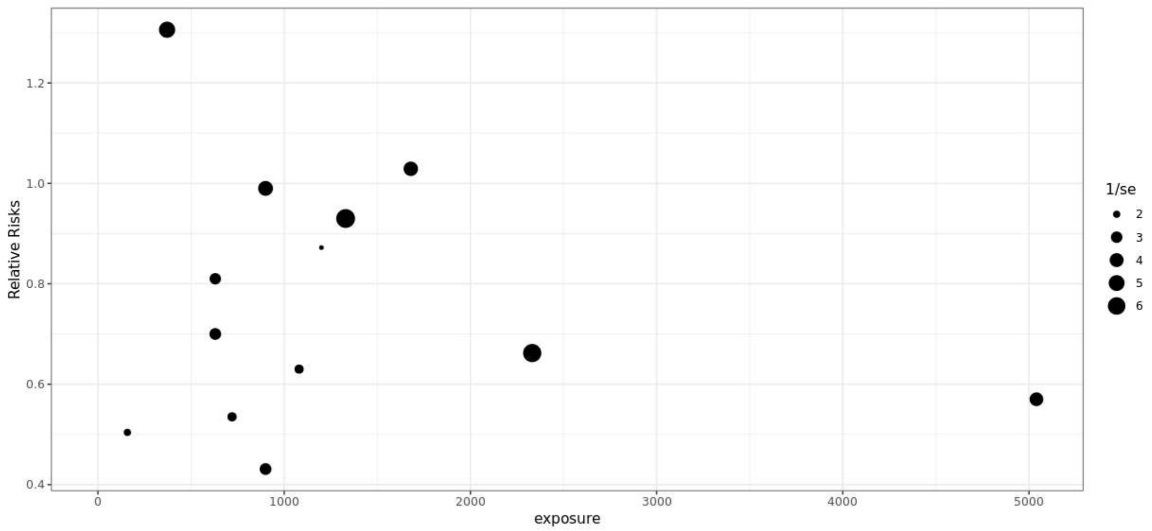

Linear Trend

```
Call:  dosresmeta(formula = logrr ~ exposure, id = id, type = type,
    cases = cases, n = n, data = dataset(), se = se, covariance = input$pscorr)

Two-stage random-effects meta-analysis
Estimation method: REML
Covariance approximation: Greenland & Longnecker

Chi2 model: X2 = 14.3032 (df = 1), p-value = 0.0002

Fixed-effects coefficients
             Estimate  Std. Error        z  Pr(>|z|)  95%ci.lb  95%ci.ub
(Intercept)   -0.0002      0.0000  -3.7820    0.0002   -0.0003   -0.0001  ***
---
Signif. codes:  0 ‘***’ 0.001 ‘**’ 0.01 ‘*’ 0.05 ‘.’ 0.1 ‘ ’ 1

Between-study random-effects (co)variance components
  Std. Dev
    0.0000

Univariate Cochran Q-test for residual heterogeneity:
Q = 8.2459 (df = 7), p-value = 0.3114
I-square statistic = 15.1%

8 studies, 8 values, 1 fixed and 1 random-effects parameters
  logLik       AIC       BIC
```

```
45.5563  -87.1126  -87.2208
```

Graphical prediction

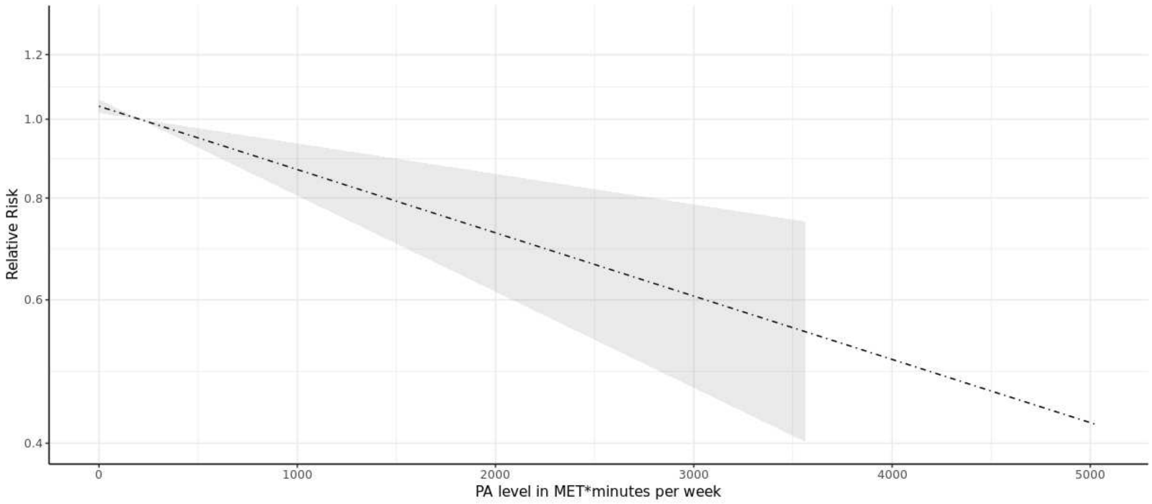

Analytical predictions

PA exposure	pred.lin	ci.lb.lin	ci.ub.lin
203.64	1.00	1.00	1.00
916.36	0.88	0.82	0.94
1934.55	0.73	0.62	0.86
2952.73	0.61	0.47	0.79
3970.91	0.51	0.36	0.72
4989.09	0.42	0.27	0.66

Models including studies with 3 or more PA exposure levels (VD)

Scatter plot of RRs vs original exposure variable

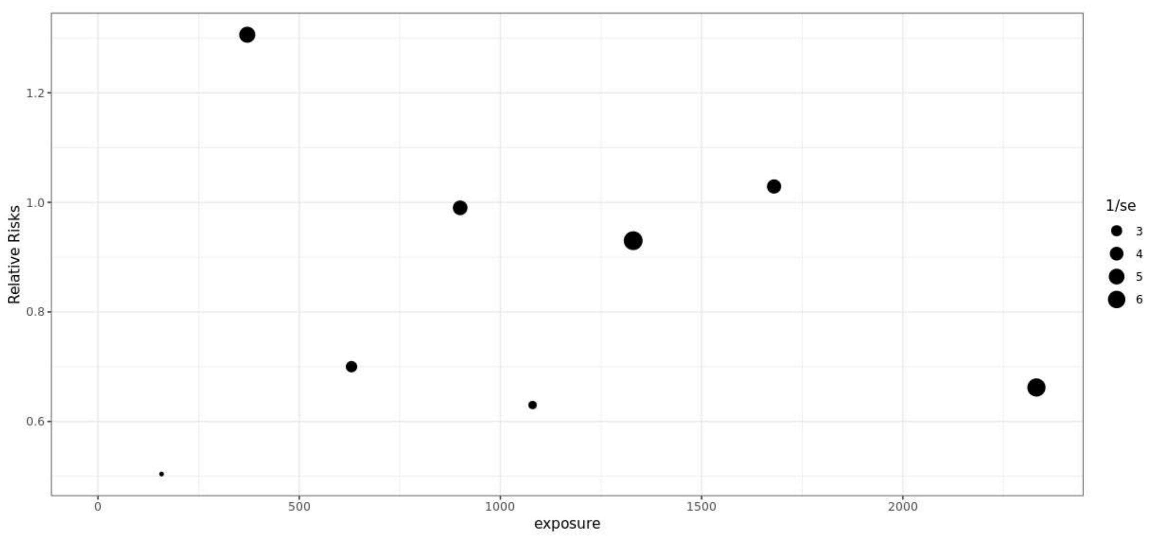

Linear Trend

```
Call:  dosresmeta(formula = logrr ~ exposure, id = id, type = type,
    cases = cases, n = n, data = dataset(), se = se, covariance = input$pscorr)


Two-stage random-effects meta-analysis
Estimation method: REML
Covariance approximation: Greenland & Longnecker


Chi2 model: X2 = 7.8907 (df = 1), p-value = 0.0050


Fixed-effects coefficients
             Estimate  Std. Error        z  Pr(>|z|)  95%ci.lb  95%ci.ub
(Intercept)   -0.0002      0.0001  -2.8090    0.0050   -0.0004   -0.0001  **
---
Signif. codes:  0 ‘***’ 0.001 ‘**’ 0.01 ‘*’ 0.05 ‘.’ 0.1 ‘ ’ 1


Between-study random-effects (co)variance components
  Std. Dev
    0.0000


Univariate Cochran Q-test for residual heterogeneity:
Q = 1.1021 (df = 2), p-value = 0.5764
I-square statistic = 0.0%


3 studies, 3 values, 1 fixed and 1 random-effects parameters
  logLik       AIC       BIC
```

```
 14.2687  -24.5373  -27.1510
```

Spline model

```
Call:  dosresmeta(formula = logrr ~ rcs(exposure, knots), id = id, type = type,
    cases = cases, n = n, data = dataset(), se = se, covariance = input$pscorr)

Two-stage random-effects meta-analysis
Estimation method: REML
Covariance approximation: Greenland & Longnecker

Chi2 model: X2 = 8.1864 (df = 2), p-value = 0.0167

Fixed-effects coefficients
                                          Estimate  Std. Error        z
rcs(exposure, knots)exposure.(Intercept)    -0.0001      0.0003  -0.1959
rcs(exposure, knots)exposure'.(Intercept)   -0.0001      0.0003  -0.5438
                                          Pr(>|z|)  95%ci.lb  95%ci.ub
rcs(exposure, knots)exposure.(Intercept)     0.8447   -0.0006    0.0005
rcs(exposure, knots)exposure'.(Intercept)    0.5866   -0.0007    0.0004
---
Signif. codes:  0 ‘***’ 0.001 ‘**’ 0.01 ‘*’ 0.05 ‘.’ 0.1 ‘ ’ 1

Between-study random-effects (co)variance components
                              Std. Dev                          Corr
rcs(exposure, knots)exposure     0.0000  rcs(exposure, knots)exposure
rcs(exposure, knots)exposure'    0.0000                       -0.9995

Univariate Cochran Q-test for residual heterogeneity:
Q = 1.5369 (df = 4), p-value = 0.8201
I-square statistic = 0.0%

3 studies, 6 values, 2 fixed and 3 random-effects parameters
  logLik       AIC       BIC
 24.6521  -39.3042  -42.3727
```

Quadratic trend

```
Call:  dosresmeta(formula = logrr ~ exposure + I(exposure^2), id = id,
    type = type, cases = cases, n = n, data = dataset(), se = se,
```

```
    covariance = input$pscorr)

Two-stage random-effects meta-analysis
Estimation method: REML
Covariance approximation: Greenland & Longnecker

Chi2 model: X2 = 8.5347 (df = 2), p-value = 0.0140

Fixed-effects coefficients
                           Estimate  Std. Error        z  Pr(>|z|)  95%ci.lb
exposure.(Intercept)        -0.0000      0.0003  -0.0541    0.9568   -0.0005
I(exposure^2).(Intercept)   -0.0000      0.0000  -0.8025    0.4223   -0.0000
                           95%ci.ub
exposure.(Intercept)         0.0005
I(exposure^2).(Intercept)    0.0000
---
Signif. codes:  0 ‘***’ 0.001 ‘**’ 0.01 ‘*’ 0.05 ‘.’ 0.1 ‘ ’ 1

Between-study random-effects (co)variance components
               Std. Dev      Corr
exposure         0.0000  exposure
I(exposure^2)    0.0000    0.9446

Univariate Cochran Q-test for residual heterogeneity:
Q = 1.2812 (df = 4), p-value = 0.8646
I-square statistic = 0.0%

3 studies, 6 values, 2 fixed and 3 random-effects parameters
  logLik       AIC       BIC
 40.3790  -70.7581  -73.8266
```

Graphical prediction

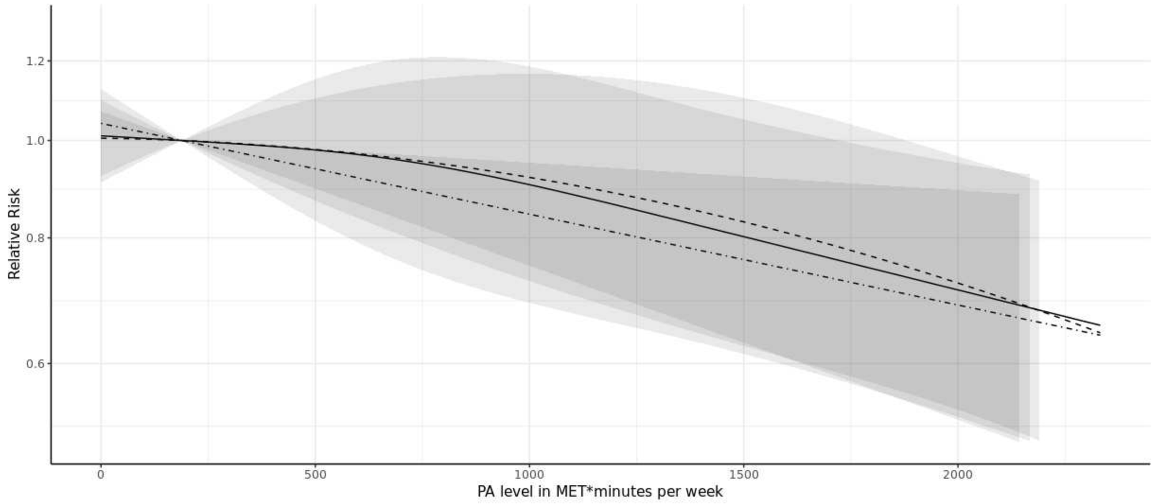

Linear Trend (dot-dash); Spline Model with knots at 20 and 80% of distribution (solid); Quadratic Trend (dashed)

Analytical predictions

PA exposure	pred.lin	ci.lb.lin	ci.ub.lin	pred.spl	ci.lb.spl	ci.ub.spl	pred.quadr	ci.lb.quadr	ci.ub.quadr
188.48	1.00	1.00	1.00	1.00	1.00	1.00	1.00	1.00	1.00
424.00	0.95	0.92	0.99	0.98	0.87	1.12	0.99	0.90	1.08
895.11	0.86	0.78	0.96	0.92	0.71	1.20	0.93	0.75	1.16
1366.22	0.78	0.66	0.93	0.83	0.63	1.08	0.86	0.65	1.13
1837.33	0.71	0.56	0.90	0.74	0.56	0.98	0.76	0.57	1.01
2308.44	0.64	0.47	0.88	0.66	0.48	0.91	0.65	0.48	0.88

References

1. Blond K, Brinkløv CF, Ried-Larsen M, Crippa A, Grøntved A. Association of high amounts of physical activity with mortality risk: a systematic review and meta-analysis. Br J Sports Med. 2020 Oct 1;54(20):1195–201.
2. Walpole SC, Prieto-Merino D, Edwards P, Cleland J, Stevens G, Roberts I. The weight of nations: an estimation of adult human biomass. BMC Public Health. 2012 Jun 18;12(1):439.
3. Crippa A, Orsini N. Multivariate Dose-Response Meta-Analysis: The dosresmeta R Package. J Stat Softw. 2016 Aug 16;72:1–15.
4. Crippa A, Orsini N. Multivariate Dose-Response Meta-Analysis [Internet]. Multivariate Dose-Response Meta-Analysis. [cited 2021 Dec 22]. Available from: http://alessiocrippa.com/shiny/dosresmeta/
